# Measuring objectification through the Body Inversion Paradigm: Methodological issues

**DOI:** 10.1371/journal.pone.0229161

**Published:** 2020-02-19

**Authors:** Cristina Zogmaister, Federica Durante, Silvia Mari, Franca Crippa, Chiara Volpato

**Affiliations:** Department of Psychology, University of Milano-Bicocca, Milan, Italy; University of Bologna, ITALY

## Abstract

Objectification occurs when a person is perceived and/or treated like an object. With the present work, we overview the available measures of objectification and present a series of studies aimed at investigating the validity of the task of inverted body recognition proposed by Bernard and colleagues (2012), which might potentially be a useful cognitive measure of objectification. We conducted three studies. Study 1 (*N* = 101) is a direct replication of Bernard et al.’s study: participants were presented with the same photos of sexualized male and female targets used in the original research. Study 2a (*N* = 100) is a conceptual replication: we used different images of scantily dressed male and female models. Finally, in Study 2b (*N* = 100), we investigated a boundary condition by presenting to participants photos of the same models as in Study 2a, but fully dressed and non-sexualized. Using mixed-effects models for completely-crossed classified data structures, we investigated the relationship between the inversion effect and the stimulus’ asymmetry, sexualization and attractiveness, and the perceivers’ self-objectification, sexism, and automatic woman-human association. Study 1 replicated the original results, showing a stronger inversion effect for male photos. However, no difference between male and female stimuli emerged in either Study 2a or 2b. Moreover, the impact of the other variables on the inversion effect was highly unstable across the studies. These aspects together indicate that the inversion effect depends on the specific set of stimuli and limits the generalizability of results collected using this method.

## Measuring objectification through the inversion paradigm: Methodological issues

On first approach, the meaning of objectification is straightforward: It refers to all circumstances under which a person is treated like an object [1]. Under these circumstances, the person may be denied autonomy and subjectivity, considered instrumental, fungible, violable, ownable by others [1] reduced to body and appearance, or silenced [2]. Over the last decade, many empirical and theoretical works have addressed the phenomenon of objectification and, more specifically, of sexual objectification, which is also the focus of the present work. Nevertheless, we still need to solve important issues, the most important of which is probably how to methodologically address this phenomenon (see [3]).

An overview of the measures used so far to investigate the sexual objectification is given below, to provide operational definitions, which help better specify what exactly researchers mean by objectification. This is important because, to draw correct conclusions from empirical data and appropriately expand our knowledge on objectification, we need to know what exactly we are measuring in our studies. Next, we will describe our investigation of the Inverted Body Recognition Task (IBRT) that was proposed by Bernard and colleagues [4, 5] (see [6]) as a proof that “perceivers may view sexualized women as objects and sexualized men as persons at a basic cognitive level” ([4] p. 469).

The aim of this work is to assess the construct validity of this specific task (i.e., IBRT) to measure women’s sexual objectification. In other words, our approach is strictly methodological, concerned one specific task applied to the investigation of one precise psychological phenomenon (i.e., sexual objectification).

### Measures of objectification

Sexual objectification is a phenomenon with many cognitive and behavioral expressions that has consequently been investigated from various viewpoints. Initial research studied the causes and consequences of self-objectification [7, 8] (for reviews, see [9, 10]), mainly through self-report questionnaires and registration of its behavioral consequences (for a review, see [11]). Recently, the focus of the research has broadened to encompass interpersonal objectification [12–14]. Like self-objectification, objectification of others has been investigated through self-report scales. In the Interpersonal Sexual Objectification Perpetration Scale [15], for instance, respondents indicate how often they engage in various behaviors of body evaluation and sexual harassment. In the Objectification of Others Questionnaire [16], participants are provided with attributes related to physical appearance (e.g., attractiveness, weight) and physical competence (e.g., health, fitness level) and are asked to rank their importance with respect to the body of other women and men. Although these measures have proven useful in research (e.g., [15, 17]), they have two major limitations: They require participants’ awareness of their own objectifying behaviors and cognitions and their willingness to report them without distortion. For instance, in the Interpersonal Sexual Objectification Perpetration Scale, participants are asked questions such as, “How often have you perpetrated sexual harassment (on the job, in school, etc.)?” Valid responses require that the respondents recognize certain behaviors as sexual harassment and are willing to respond honestly. Similarly, we cannot take for granted that when answering the Objectification of Others Questionnaire, people are aware of the importance they give to different attributes [18].

These limitations of self-reports can be circumvented by directly assessing objectification while it takes place. This can be accomplished with two classes of measures: those assessing decreased attribution of human characteristics and those assessing a focus on sexual body parts. With the first class of measures, it has been shown that after focusing on the physical appearance of a woman—instead of focusing on her as a person—both men and women ascribed her less competence and lower levels of traits typical of human nature [19]. Similarly, focusing on appearance decreased ascriptions of warmth, competence and morality to a woman (but not to a man [20]). Moreover, when presented in a sexualized manner, both women and men were judged as possessing lower degrees of mental states, intelligence, and morality [21] (see also [22, 23]). These latter results are in line with objectification theory’s claim that sexualization is a major cause of objectification [7].

The second class, which encompasses a more heterogeneous group of measures, is grounded on the notion that sexually objectified individuals are reduced to their bodies and especially to the sexual body parts [7]. Researchers have studied the focus of attention on sexual body parts by monitoring gaze through eye tracking [24], the dot-probe task [23], and the part-versus-whole body recognition paradigm [25]. Results show that when women are presented in a sexualized way, perceivers direct more attention to their bodies [23, 26]; furthermore, images of women—but not images of men—suffer enhanced attention to the body parts as compared to the whole body [25]. Measures of reduced ascription of human characteristics and of increased focus on sexual body parts are grounded on the unspoken assumption of an objectification continuum, which ranges from regarding a target individual as completely human to regarding her as an object and denying her humanity. The term ‘objectification’ is used as a metaphor, signifying that people at times are attributed *less* humanity and are treated or perceived more *like* objects (see, e.g., [13]).

Bernard and colleagues [4], however, proposed a task based on the recognition of persons’ photos presented right-side-up and upside-down, which for simplicity we call Inverted Body Recognition Task (IBRT). Based on evidence collected through the IBRT, they claimed that sexualized women are cognitively processed *in the same way as* objects. Such a strong claim, if true, would make objectification more than a metaphor, and it would imply a complete denial of human nature. Not surprisingly, Bernard et al.’s work has immediately attracted both considerable interest and criticism [27, 28]. Because of the theoretical importance of the authors’ claim, and given that the IBRT might be a useful cognitive measure of objectification, we deemed it important to thoroughly investigate its construct validity and the elements of concern that have been raised.

### The Inverted Body Recognition Task

The IBRT aims at measuring the inversion effect, which was initially observed for faces [29] and, subsequently, for body silhouettes [30]. The inversion effect is an impaired recognition of stimuli presented upside-down, as compared to those presented right-side up. Most objects are more difficult to recognize upside-down, but this effect is stronger for human faces [30]. This was initially interpreted as evidence that face recognition involves unique cognitive processes and that perception of faces upside-down interferes with these unique processes (but see [31]). The inversion effect has been investigated for faces and, less extensively, also for body shapes. In the recent literature, it is commonly explained through the distinction between configural and analytical processing. Configural processing is the processing of the spatial relations between features of a complex stimulus (for example, the relative position of eyes and nose in a face). Analytical processing takes place when the elements that compose a stimulus are considered, ignoring the relations among them. Analytical processing is thought to be largely unaffected by vertical stimulus inversion (but see [32]). The absence of inversion effects, with upside-down stimuli recognized as easily as right-side up, would therefore indicate less configural processing. Body silhouettes and faces would suffer stronger inversion effects than other objects because, typically, they are configurally processed. It is, however, important to notice that few studies investigated the body inversion effect (BIE), and scholars are very cautious in explaining the mechanisms underlying this phenomenon. Some argue that human bodies may “represent a unique stimulus class with specialized processing mechanisms, which differ from face and object processing” ([33] p. 873]. Others insist that “the extensive understanding that we have about mechanisms that may underlie the face inversion effect may not be necessarily applicable to account for the BIE, until further research is completed with human bodies” ([34] p. 766]. Some others contend that the head posture plays a central role in explaining the BIE (e.g., [35]).

Bernard and colleagues [4, 5] proposed using the inversion effect to investigate objectification, assuming that when a person is perceived as a human being, she should be processed configurally, while she should be processed analytically when perceived as an object. They argued that “If sexualized women are viewed as objects and sexualized men are viewed as persons, then sexualized female bodies will be recognized equally well when inverted as when upright (object-like recognition), whereas sexualized male bodies will be recognized better when upright than when inverted (person-like recognition)” ([4] p. 469).

**Fig 1:**
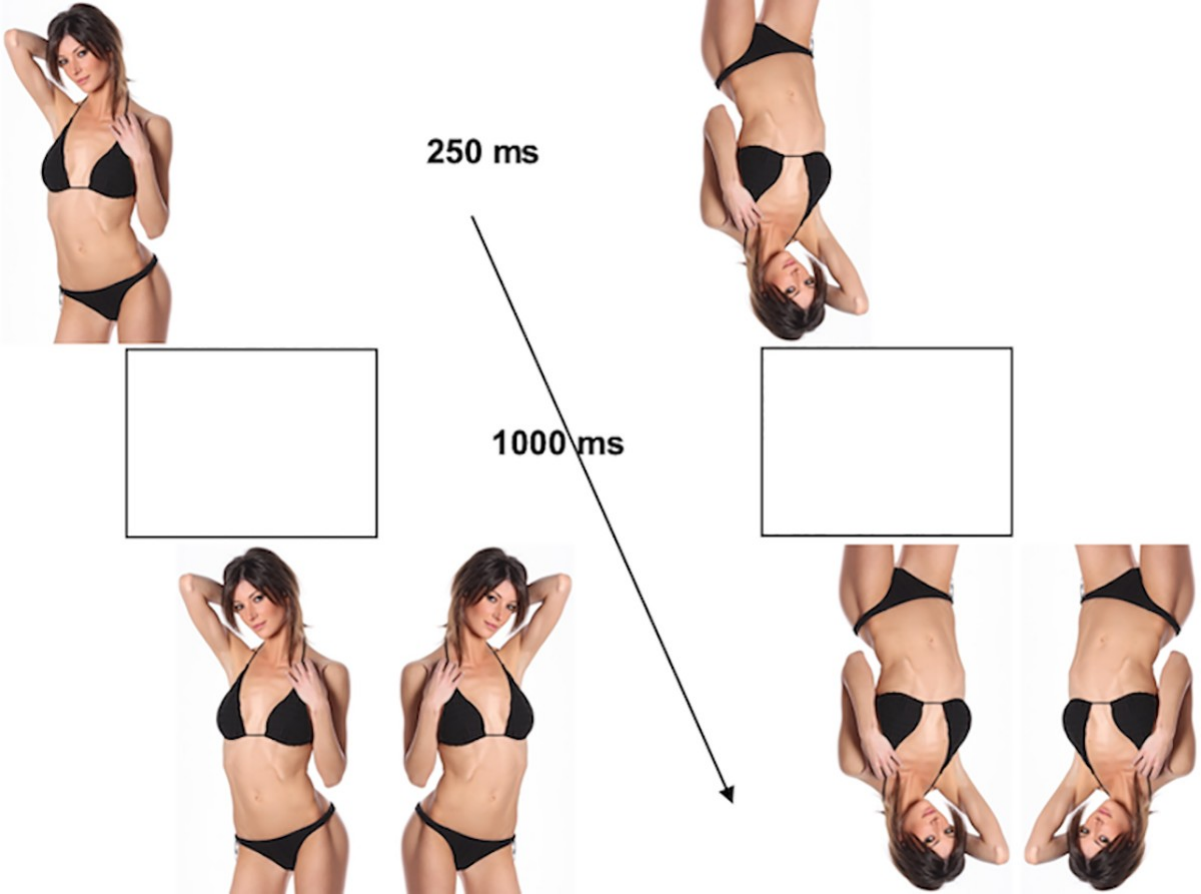
Typical trials in the Inverted Body Recognition Task (the pictures shown are for illustrative purpose only).

In Bernard’s IBRT, participants are presented with photographs of individuals for a recognition task. In each trial, a photo is presented at the center of the monitor for 250 ms, followed by a blank screen for 1000 ms, after which two stimuli are presented side by side: the same photo and its left-right mirror image. Participants must indicate which of the two stimuli was presented beforehand. Half of the photos are presented right-side up, half upside-down. The orientation is the same in the initial exposure and in the recognition phase of each trial. Fig 1 shows two typical trials of the task. The main dependent variable is the accuracy of response. Reaction times must be inspected to check that differences in accuracy are not due to differences in inspection times [28] (see also [36]).

Bernard and colleagues [4] presented the IBRT to a sample of 78 participants, using a total of 48 sexualized photos, half of women and half of men, half right-side up and half upside-down. Participants correctly recognized a lower proportion of upside-down as compared to right-side up photos of males, whereas their recognition rate for female photos was not influenced by orientation. In other words, the photos of sexualized men suffered the inversion effect, but those of sexualized women did not. The authors considered these results to be evidence that sexualized men were perceived as humans and sexualized women as objects. Subsequent evidence that the inversion effect was stronger for male photos, although it was also present for female photos ([5] Study 1; [37]), was interpreted as showing that sexualized men elicited less objectification compared to sexualized women [5].

At first glance, the reasoning is straightforward: Based on the premises that (a) the presence of inversion effects is a signature of configural processing, and (b) configural processing indicates that the stimuli are processed as human beings, Bernard and colleagues [4, 5] conclude that (c) the lower the inversion effect, the more the stimulus is objectified. Unfortunately, neither of the premises can be taken for granted. Contrary to premise (a), object recognition is influenced by inversion (see [28]). More importantly, even feature recognition can suffer inversion [32]. Contrary to premise (b), empirical evidence also shows configural processing for objects, especially for those with which individuals have high expertise [38] (see also [39, 40]). In fact, using the IBRT, Cogoni and colleagues [41] found that totally undressed mannequins with a female body shape suffered inversion effects that were significantly stronger than those suffered by other objects (see also [42], for similar evidence of inversion effects in robots) and as strong as those suffered by completely dressed women. If we accept an interpretation of the inversion effect in IBRT as an indicator of how human-like the stimulus is perceived, based on this similarity of inversion effects for dressed women and mannequins we should conclude that mannequins with female appearance are perceived as equally human as completely dressed women.

A further problem is that many non-social factors influence the magnitude of the inversion effect [28]. In studies investigating the inversion effect in perception, simple body silhouettes are used. Bernard and colleagues, on the other hand, to investigate objectification, used human images that were considerably more complex and richer in details. Any perceptual dissimilarity between the stimuli presented to participants in different conditions is therefore a potential source of contamination. For instance, the male and female photographs used by Bernard and colleagues differed in dimensions other than gender (e.g., hairstyles, levels of asymmetry, complexity of silhouettes, number and specificity of perceptual features; see [27, 28]). The potential impact of these sources of variability makes it virtually impossible to rule out alternative explanations of differences in inversion effects between conditions characterized by the use of different stimuli.

In sum, from a theoretical standpoint, it is not clear whether the inversion effect in the IBRT measures objectification and whether its size is an unequivocal index of the extent of objectification. Let us therefore take a pragmatic stance: What empirical proofs are available for and against considering the inversion effect measured with IBRT as a measure of objectification?

### Empirical evidence for and against the IBRT as a measure of objectification

In their initial study, Bernard and colleagues [4] found a significant inversion effect for pictures of sexualized men and no effect for sexualized women. However, they presented different sets of male and female photos in the upright and in the inverted conditions. Subsequently, Schmidt and Kistemaker [27] conducted a study with the same materials, but with the important difference that—contrary to Bernard and colleagues—they counterbalanced the stimulus orientation: Participants were presented with two trials for each photograph, in one trial the photo was presented upright, in the other upside-down. Responses to the original stimulus setup provided an almost exact replication of Bernard and colleagues’ results, but the opposite pattern emerged with the counterbalanced setup, namely, an inversion effect for female stimuli and no significant effect for male stimuli. In line with Tarr’s critique [28], these results indicate that the specific stimuli may strongly impact the inversion effect.

In an attempt to investigate the effect of sexualization on the inversion effect with new stimuli, Schmidt and Kistemaker [27] created a set of male and female photos with comparable levels of asymmetry. All individuals were portrayed nude, and a less sexualized version of each photo was produced by covering the body with an opaque skin color from upper limb to upper chest. They presented all pictures interspersed in an IBRT and found no difference in the size of the inversion effect for female and male targets, either when they were presented nude or with opaque superimposition. Unfortunately, no firm conclusion can be drawn from these results for three reasons: First, nudity may alter cognitive reactions [43, 44] and could therefore cause different outcomes as compared to the sexualized, but not nude, photos of the original research. Second, the opaque superimposition prevented focalization on the sexual parts, and this, instead of the nudity, might have enhanced configural processing and the inversion effect with both male and female covered figures. Third, the presentation of covered images interspersed with the nude ones in the same task might have influenced the processing of the stimuli presented without the opaque masking. More specifically, presenting in the same IBRT half-stimuli with covered sexual body parts might have interfered with the analytical processing of the nude stimuli in at least two ways: (a) by activating an elaboration mindset that disregarded the analytic information that was present in only half of the stimuli, and (b) through order effects, namely, by the influence of earlier experience with one covered stimulus on subsequent elaboration of the same nude stimulus.

Indeed, with a similar manipulation, Bernard and colleagues ([5], Study 1 and Study 2a) found very different results. Using their original sexualized materials, in Study 1, the authors found stronger inversion effects for male than female photos, but when they pixelated the sexual body parts in their Study 2a, the size of the inversion effect was similar for male and female pictures.

Another set of experiments that used the IBRT with new sets of stimuli is the research by Cogoni and colleagues [41], mentioned above. In particular, in Study 2, they presented participants with images of sexualized women, non-sexualized women, and mannequins. The authors did not report the test of the difference in the inversion effect between sexualized and non-sexualized women. In line with the theoretical expectation that non-sexualized women should be perceived as more human than sexualized ones, in their data the inversion effect is stronger in size in non-sexualized as compared to sexualized women. However, contrary to what we would expect based on the fact that mannequins are not human, the inversion effect is present for mannequins as well, and the difference between the accuracy for upright and inverted images is twice as big for mannequins, as compared to non-sexualized women. In Study 3, data on accuracy are not very informative due to the presence of ceiling effects in most conditions, with estimates of accuracy at .97 or above. In Study 4, in which both male and female sexualized and non-sexualized pictures are presented, data on accuracy, again, show only a main effect of orientation.

It is indisputable that the *specific* characteristics of the stimuli play a role in the inversion effect. Salient elements (e.g., clothing, visible body parts) might direct attention to the features versus configurations. The difference between female and male target recognition might be due to a higher distinctivity of female body parts: This would explain why pixelating them leads to increased similarity in body inversion effects for male and female stimuli [5]. Therefore, even seemingly small differences between conditions, such as pixelation or opaque masking, should be avoided, because such differences might alter perception processes. The perceptual characteristics potentially influencing the inversion effects are infinite. Therefore, compelling proof for the validity of the IBRT as a measure of objectification would require a comparison of the scores obtained when presenting identical materials to participants assigned to different conditions (e.g., by manipulating instructions) or to participants characterized by individual differences in variables associated with objectification. To our knowledge, only one study has used this strategy: Bernard and colleagues [5] (Study 3) administered to participants an IBRT with photos of sexualized women. Half of the participants were provided with humanizing information about these women, while the other half were not. The results indicated an inversion effect with humanizing instructions, while no inversion effect emerged without such instructions.

Civile and Obhi [45] used a different inversion paradigm, in which participants were initially presented with an original set of pictures of sexualized women and men either upright or upside-down, and they were subsequently asked to perform an old/new recognition task. Interestingly, they showed that when they had been primed with the concept of power, both male and female participants showed the inversion effect for targets of their own sex, but they did not show any inversion for participants of the opposite sex. It is worth mentioning that in Civile and Obhi’s Study 1, there was also a neutral condition (i.e., no priming of sorts), and the authors found an inversion effect for male but not for female targets. Civile, Rajagobal, and Obhi [46] replicated and extended their finding, namely, Caucasian participants primed to high-power did not show an inversion effect for Caucasian sexualized models of the opposite gender, but they did for Caucasian targets of their own gender and for both male and female sexualized Asian models.

More recently, Xiao, Li, Zheng and Wang [47] carried out two studies. In Study 1, participants were initially primed with high-power, low-power or no power (control condition). Then, they performed the IBRT, same task and stimuli used by Bernard and colleagues [4, 5]. In Study 2, the authors used the modified version of the paradigm by Civile and collaborators [45, 46], with a new set of stimuli, and primed participants with high- versus low-power. Results showed that that in the control condition in Study 1 (which might be considered similar to a direct replication of [4]), and in both conditions of power in Study 2, recognition of sexualized male as well as sexualized female targets suffered the inversion effect.

All things considered, the existing evidence on the validity of the IBRT is inconclusive. Alongside results showing that the inversion effect for female photos is increased by stimulus alterations aimed at decreasing sexualization ([5], Study 2; but see [27]) and by individuating instructions ([5], Study 3), other evidence shows that even objects with a human shape suffer inversion effects [41]. Given its potential theoretical importance as a proof that sexualized women are not just perceived *like* objects in a metaphorical way but *as* objects at the cognitive level, and given its possible utility as a measure of objectification, we aim to replicate Bernard et al.’s study and analyze the role of asymmetry in their materials, which was claimed to be a critical issue but never demonstrated as such ([27, 28, 41]; but see [48]). Moreover, we aim to investigate the role of social variables thought to affect objectification. This same analysis will be conducted with new photographs characterized by controlled levels of asymmetry and sexualization. With this work, we hope to provide the scientific community with information on the utility of IBRT for the study of objectification.

### The present research

We conducted three studies: a direct replication of Bernard and colleagues’ study [4], using their original materials, but with counterbalanced stimuli as in [5] (Study 1); a conceptual replication–to our knowledge the first one–with different sexualized materials to investigate whether the same pattern of results would emerge with different stimuli (Study 2a; indeed, so far most of the evidence in favor of the IBRT has emerged from research using the set of stimuli developed by Bernard and colleagues [4]); an investigation of the inversion effect with non-sexualized stimuli (Study 2b).

We expected (H1) stronger inversion effects for male than female targets and (H1b) that the participant’s gender would not moderate this difference between male and female targets. The confirmation of this hypothesis in Study 1 would be a direct replication of [5], and its confirmation in Study 2a would be a conceptual replication, enhancing its external validity beyond the specific stimuli. Study 2b tested a boundary condition, namely, whether differences in inversion effects between male and female targets would emerge also for non-sexualized stimuli.

We further reasoned that if the absence of an inversion effect is an outcome of objectification, it should be empirically related to a series of characteristics of the target stimulus and of the perceiver. Therefore, we investigated the relationship between a series of variables pertaining to the stimuli and the perceiver, and the size of the inversion effect, to gather new evidence on the construct validity of the IBRT as an indicator of objectification.

#### Target stimulus variables

Asymmetry. As noted by Tarr [28] and Schmidt and Kistemaker [27], differences in the asymmetry of stimuli could cause methodological artifacts in inversion effects. Schmidt and Kistemaker [27] showed that the male and female stimuli used by Bernard et al. [4, 5] were significantly different in asymmetry. Cogoni and colleagues [41] recently attempted to investigate the role of asymmetry in the IBRT. They compared four different categories of stimuli (fully dressed and scantily dressed women, mannequins, houses; Studies 1–2), differing in average level of asymmetry, and found no evidence that asymmetry mediated the relation between category of stimuli and inversion effect. In their Study 3, they created two sets of stimuli (i.e., high and low in asymmetry) and found that when asymmetry was high, no difference emerged in the inversion effect for fully and scantily dressed stimuli; when asymmetry was low, images of fully dressed women were characterized by a higher inversion effect than scantily dressed ones. In sum, asymmetry might play a role in the inversion effect. Importantly, no study before had directly tested the alleged impact of the difference in asymmetry between female and male stimuli on the original set of images by Bernard and colleagues [4]. Using a mixed-effects model approach, we were able to directly investigate the impact of asymmetry on the inversion effect, and test whether the inversion effect would still be present, when asymmetry was statistically controlled.

Based on the literature reviewed above, we hypothesized that asymmetry would decrease inversion effects (H2).

Sexualization is considered an important cause of objectification [49, 50] that could more strongly impact women’s objectification for both evolutionary and cultural reasons [50]. Following Fasoli, Durante, Mari, Zogmaister, and Volpato [51], we measured each photo’s objective level of sexualization through Hatton and Trautner’s [52] scale, which defines photo sexualization as a combination of sex cues (e.g., posture, nudity, face expression). We hypothesized that target sexualization would decrease inversion effects (H3).

Attractiveness is important for social interactions [53]. In our society, attractiveness is strongly intertwined with sexualization. While sexualization was measured as an objective characteristic of the stimulus, for attractiveness, we measured subjective evaluations of the perceivers. Riemer and colleagues [54] found that greater perceived attractiveness was associated with an increased objectifying gaze: Participants gazed shorter to the faces, but longer to the chests and waists of the more attractive women. This suggests that greater attractiveness might be associated with higher levels of objectification. Therefore, we hypothesized that target attractiveness would decrease inversion effects (H4).

#### Perceiver variables

Automatic Woman-Human Associations were measured because we reasoned that if the IBRT captures spontaneous objectification of women, it should be negatively related to the degree to which ‘women’ are associated with ‘humanity’ in automatic cognition. To this aim, we measured the woman-human (vs. object) association through a single-category IAT (SC-IAT, [55]; see [50]). We hypothesized that participants with higher woman-human SC-IAT scores would show stronger inversion effects, especially for female targets (H5).

Self-objectification might prove particularly important. A relationship between self-objectification and objectification of others has emerged in self-report studies [16], and may be due to the internalization of societal norms regarding appearance standards. Inconclusive results come from a study by Bernard and colleagues [56]. The authors, through a “whole body/body part” paradigm, found that self-objectification was negatively associated to recognitions of whole bodies among high self-objectifiers, but it was not to the recognition of body parts. Recently, Groves, Kennett, and Gillmeister [57] found evidence suggesting that, in adolescents, high self-objectification and body image concerns and body image disturbance, might be associated with lower inversion effects on a body inversion task. Therefore, we measured self-objectification using the Objectified Body Self-Consciousness scale (OBCS, [58]; see also [59, 60]). We specifically investigated whether body surveillance (BSV, the tendency to think of one’s own body in terms of how it looks rather than how it feels) and body shame (BSH, the belief of being a bad person when not achieving cultural body standards) would moderate the inversion effect. BSV is considered as a behavioral indicator of self-objectification [11] and has been related to various behavioral consequences (see [61]). As Western culture proposes beauty ideals that are virtually impossible to meet, BSH is thought to be one common consequence of the internalization of Western ideals regarding body appearance [62]. Increased levels of BSH, therefore, signal a higher endorsement of the objectifying culture. Consequently, we hypothesized that respondents with high self-objectification scores on the BSH and BSV subscales of OBCS would show weaker inversion effects (H6).

Sexism has been suggested to be positively related to the objectification of women [63, 64]. We used the Ambivalent Sexism Inventory (ASI) and the Ambivalence Toward Men Inventory (AMI) [65–67] to measure sexist attitudes and gender beliefs, differentiating their benevolent and hostile components. We hypothesized that participants with higher sexism scores would show lower inversion effects (H7).

We investigated all of these hypotheses of moderation both with sexualized (Study 1 and Study 2a) and non-sexualized body images (Study 2b), because it is possible that for sexualized stimuli, the social variables have less impact, as their high sexualization might be a potent cause of objectification, concealing the influence of other variables. If so, only non-sexualized materials would provide the opportunity for the observation of moderating effects.

We also examined the inversion effect using images of tall buildings to compare the effects observed for human targets with those observed for non-human targets. We chose tall buildings because, similarly to humans, we have a long learning experience of seeing buildings in an upright position, we cannot easily put them upside-down, and their vertical axis is longer than the horizontal one. We specifically tested (Q1) whether images of tall buildings suffered inversion effects comparable to the inversion effects for photos of individuals, and (Q2) the impact of asymmetry on the inversion effect for buildings.

By and large, our strategy aimed at providing the best opportunities for validation of the IBRT.

## General method

We measured the asymmetry and sexualization of each photo of the original material [4] and of the new material developed for the present research. We subsequently conducted three studies.

We created a continuous index of asymmetry based on eight body parts (forehead, chin, navel, eyes, shoulders, elbows, thighs, hands). For unique points (e.g., navel), we measured the Cartesian distance from the vertical line in the center of the image, whereas, for double points (e.g., eyes), we measured the Cartesian distance between the right point and the mirror image of the left point. All measures were taken in pixels. We summed the eight distance measures to form a single index (inter-rater reliability: α = .98 for the original set, α = .97 for the new sets of photos). Full information on the asymmetry index and the SPSS syntax to compute it are provided in S1 File.

As noted, the three studies differed only for the materials used in the IBRT. Importantly, the data for Studies 2a and 2b were collected in parallel in the same laboratories by the same experimenters, allowing us to directly compare the results. Sample sizes were determined based on the initial study of Bernard and colleagues [4], which consisted of 74 participants. For the sake of clarity, we first describe the common methodological aspects of the studies, and then we report the results of each study separately. We report all measures, manipulations, and exclusions in these studies.

### Participants

One-hundred and one participants (52 females, 49 males, age 19–29, *M*age = 22.67, *SD* = 2.44, 97 Italian, 4 of other nationalities) took part in Study 1. One-hundred participants (51 females, 49 males, age 19–32, *M*age = 22.74, *SD* = 2.55, 99 Italian, 1 of another nationality) took part in Study 2a. One-hundred participants (50 females, 49 males, 1 missing value, age 18–45, *M*age = 23.06, *SD* = 3.80, 95 Italian, 4 of other nationalities, 1 missing value) took part in Study 2b. All participants but 7 were university students and received credit for participation.

### Procedure

Each study was presented as a research on opinions toward women, men, and their relationships on the cognitive elaboration of images of women, men, and other stimuli. After providing written informed consent, participants were administered the IBRT and the inverted building recognition task. Then, they performed the SC-IAT and answered the ASI and AMI inventories, with their items intermixed in a fixed random order, and, subsequently, the OBCS in a fixed random order. Upon completion of the questionnaires, participants were presented again with the IBRT target photos, right-side up, one at a time, in random order, and evaluated their attractiveness on a 5-point scale (1 = *not at all attractive*; 5 = *extremely attractive*). Finally, they indicated their gender, age, nationality, and sexual orientation, received a brief explanation of the research, and the experimenter answered their questions. The entire experiment took approximately 30 minutes.

### Stimuli

#### Original materials

This set consisted of the 48 photos of sexualized individuals (24 men and 24 women) used by Bernard et al. [4], with a standardized size of 500*750 pixels on a white background. As indicated in Table 1, the mean level of asymmetry in the original set was higher for photos of women than of men, *t*(46) = 3.78, *p* < .001, Cohen’s *d* = 1.09. The mean level of sexualization in the original stimulus set was not significantly different for male and female pictures, *t*(46) = 0.75, *p* = .45, Cohen’s *d* = 0.22.

**Table 1:** Main descriptive statistics for asymmetry and sexualization scores of the images. All studies.

Type of images	*N*	Asymmetry				Sexualization			
		*Min*	*Max*	*M*	*SD*	*Min*	*Max*	*M*	*SD*
**Study 1**									
Men	24	55.60	572.47	275.30	123.16	5.00	12.00	8.54	1.59
Women	24	226.84	979.78	477.06	231.01	5.50	12.50	8.83	2.13
**Study 2a**									
Men	12	101.75	517.36	257.23	114.35	7.00	9.50	7.79	0.81
Women	12	116.83	500.40	240.74	130.04	5.00	12.00	6.67	2.13
**Study 2b**									
Men	12	119.41	517.36	224.44	106.83	0.00	2.50	1.08	0.76
Women	12	70.80	627.15	230.88	165.74	0.50	5.00	2.29	1.28
**All studies**									
Buildings^a^	24	111.10	1374.66	441.61	274.78				

^a^ The same images of buildings were used in all studies (Study 1, 2a and 2b).

#### New materials

To create a more controlled but still comparable new sets of stimuli, we made our selections from the Internet, but the sexualized and non-sexualized images portrayed the same individuals. More specifically, for each of 12 different female and 12 male models we selected a fully dressed photo and one in which the model wore underwear or a swimsuit, and we rescaled them to 500*750 pixels. All models gazed directly at the camera and appeared on a white background. A one-way ANOVA on the asymmetry of the photos, with sex and clothing as factors, showed no significant effects, *F* < 1. This showed that, dressed and undressed, male and female targets did not differ in terms of asymmetry (see S1 File for the complete procedure of stimulus selection and asymmetry measurement). As illustrated in Table 1, the images of new dressed targets were rated as less sexualized than the images portraying the same undressed individuals, *t*(46) = 13.126, *p* < 001, *d* = 3.79. The level of sexualization in the new undressed dataset was lower than that in Bernard et al.’s dataset, *t*(70) = 3.223, *p* = .002, *d* = 0.82, but all undressed stimuli fell within the “sexualized” as defined by Hatton and Trautner [51, 52]. To sum up, the new materials did not differ in terms of asymmetry, but dressed and undressed images did differ in terms of sexualization. Most importantly, the same male and female models were used in both sets of stimuli. Therefore, facial and body features did not vary from Study 2a to 2b.

Stimuli, which are subject to copyright, are available for inspection upon request to the first author.

#### Images of buildings

A set of 24 images of tall buildings (e.g., skyscrapers, bell towers) was selected from the Internet, rescaled to a 500*750 pixel size with the original background replaced with a white background. We chose buildings characterized by at least some asymmetry because, otherwise, it would be virtually impossible to recognize them from specular foils, rendering the inversion score uninformative.

### Indirect measures

#### Inverted body recognition task

We followed the original IBRT procedure [4], with materials counterbalanced, as in [5]. In Study 1, participants performed two blocks of 48 recognition trials. In each trial of the first block, they saw one of the 48 different pictures from the original set for 250 ms, followed by a 1000 ms blank screen. Subsequently, the picture was presented again on the monitor side-by-side with its mirrored image for a forced-choice recognition task (see Fig 1). The second block was identical except that the orientation of the stimuli was counterbalanced. The order of blocks was counterbalanced between participants. Since Bernard and collaborators [5] registered a relatively high loss of participants due to a bad performance in the IBRT (approximately 8% overall), to prevent loss of participants due to a misunderstanding of instructions, before the actual IBRT, the participants went through a familiarization block in which they were presented with eight trials similar to the main IBRT, with four different pictures presented upside-down and right-side-up. During familiarization, the initial picture was presented for 500 ms and participants received feedback on correctness. The familiarization block was repeated until participants answered at least 75% of the trials correctly or had performed four blocks of familiarization, whichever came first. Thereafter, participants were informed that familiarization was over and were advised to address the experimenter if they had questions; otherwise, they could begin the main phase of the task.

The IBRT in Studies 2a and 2b was identical, except that participants performed two blocks of 24 trials and were presented with the scantily dressed (Study 2a) or fully dressed (Study 2b) photos from the new set.

#### Inverted building recognition task

This task consisted of two blocks of 24 trials, with the same structure as the IBRT, except that images of buildings were presented.

#### Single category IAT

Participants were presented with a standard SC-IAT with the category women and the attributes human-object. We administered to the participants the two blocks of SC-IAT in a fixed order with the woman-human association always preceding the woman-object association to decrease method-related variability [68]. We used four words (the Italian for person, individual, humanity, feelings) for the category ‘person’ and four words (the Italian for thing, object, inanimate, instrument) for the category ‘object’. For the category ‘woman’, participants were presented with five images of women that were different but had similar levels of sexualization as compared to those presented in the IBRT.

#### Strategy of analysis

In each study, we first tested the presence of the inversion effect (i.e., greater accuracy) in the recognition of photos of persons presented upwards as compared to upside-down. Next, we investigated whether Bernard and collaborators’ results [4] were replicated. We subsequently tested the impact of asymmetry, sexualization, and attractiveness (stimuli), and implicit woman-human association, self-objectification, and sexism (perceiver) variables to investigate evidence on the construct validity of the IBRT as an indicator of objectification. Descriptive statistics, correlations and reliabilities of the OBCS subscales and the ASI and AMI subscales are reported in S2 File.

Data from all three studies were analyzed as follows: We tested both the accuracy and RTs of IBRT applying, respectively, logit and linear mixed-effects models for completely-crossed classified data structures, with random intercepts for both participants and targets. We adopted this strategy because the stimuli were nested within (as they were presented to) each person, and, *vice versa*, all persons were nested within each stimulus, as it was submitted to all participants [69, 70]. These models overcome several drawbacks of General Linear Models (GLMs). In the first place, their results can be generalized not only to subjects but also to items due to the simultaneous inclusion of both random factors into the same analysis. Moreover, they profit from the general advantages of mixed-effects modeling, as far as assumption on homoscedasticity or sphericity, and robustness against mixing discrete and continuous predictors are concerned. In addition, the accuracy of an answer is binary (correct or incorrect), and it is suitably cast in a logit regression, with estimation advantages over approximation of the percentage of correct recognitions.

Data analyses were performed using SPSS 25 [71], and the *glmer* function of the *lme4* package and the *lme* function of the *nlme* package in the R environment [72]. We tested a specific model for each of the hypotheses and questions outlined above, entering the relevant factors and interactions. Datasets and R syntaxes are available at OSF platform (https://osf.io/eb83j/).

As target sex and participant gender are important when dealing with women’s sexual objectification, after the main test of the impact of each moderator on the inversion effect, we further investigated potential influences of these variables with a hierarchical strategy: We initially tested whether the moderator had a general effect on inversion with a target orientation * moderator interaction. Next, we performed a test for possible differential effects of the moderator depending on target sex, with a target orientation * moderator * target sex interaction.

To leave no stone unturned, we also conducted a check for differential effects involving both target sex and participants gender, with a target inversion * moderator * target sex * participant gender interaction. When a moderator showed a 3-way interaction with target orientation and target sex or a 4-way interaction with target orientation, target sex and participant gender, we tested its interaction with target orientation in each of the four conditions created by target sex * participant gender. Because the results of these additional analyses were uninformative, as no pattern emerged, for the sake of clarity and brevity we reported them in S3 File.

Here, we focus our report on accuracy results, as accuracy so far has been considered the primary outcome in the research on the IBRT. Full details on the analyses performed on both accuracy and latency are presented in the S3 File.

All continuous predictors were mean centered before the analysis. Dichotomous predictors were dummy coded as follows: target orientation 0 = inverted, 1 = upright; Target Sex 0 = male, 1 = female; Participant Gender 0 = female, 1 = male. Therefore, we expected a positive impact of target orientation on accuracy, which indicates a more accurate performance for upright stimuli. Consequently, the impact of moderating variables on the inversion effect as measured by accuracy should be interpreted as follows: A positive regression coefficient of the interaction between a moderator and target orientation indicates that the moderator enhances the inversion effect (it increases the accuracy of the upright images as compared to those upside-down), while a negative sign indicates that the variable decreases the inversion effect.

In the analyses, from all our samples, we first excluded outliers based on recognition scores and mean reaction times in the IBRT (less than 75% correct or an average response latency above 3000 ms; 4 participants in Study 1, 12 in Study 2a, 6 in Study 2b. Further, following Bernard and colleagues [5], and considered all scores that deviated more than three median absolute deviations from the median as potential outliers. We used this same criterion to identify potential outliers for all variables in the study. Given our main focus on the IBRT, data were listwise excluded if they were outliers on IBRT, while they were pairwise excluded if they presented outlying values on one measure of interindividual differences. We then excluded non-Italians to have participants with the same cultural background. Therefore, the analyses were conducted on 93 Italian participants with valid performance in the IBRT in Study 1, 87 in Study 2a, and 91 in Study 2b.

## Results

### Study 1

#### Data cleaning

Two participants responded correctly to fewer than 75% of trials in SC-IAT, so these scores were pairwise discarded for those analyses that involved the SC-IAT measure. Six participants with bad performance were identified and dropped from the analyses on the inversion effect for buildings.

The mean proportions of correct responses for the IBRT, depending on sex and orientation of the target, are presented in Table 2. A synopsis of the results is presented in Tables 3–6, left column, and a complete report of all accuracy and latency results is presented in the S3 File.

**Table 2:** Mean proport on of correct responses for the IBRT, depending on sex and orientation of the target (standard deviations in parentheses). All studies.

	Male targets	Female targets	Buildings
**Study 1**			
Right-side up	.86 (.10)	.91 (.11)	.89 (.34)
Upside-down	.79 (.12)	.88 (.11)	.87 (.32)
**Study 2a**			
Right-side up	.87 (.33)	.90 (.30)	.86 (.35)
Upside-down	.80 (.40)	.81 (.39)	.85 (.35)
**Study 2b**			
Right-side up	.89 (.31)	.88 (.33)	.88 (.35)
Upside-down	.86 (.34)	.81 (.39)	.86 (.35)

**Table 3:** Results of the hypotheses testing for the replication of inversion effects (standard deviations in parentheses). All Studies.

Hypotheses	Study 1	Study 2a	Study 2b
Effect of target orientation	*b* = 0.50 (0.06), 95% CI [.37, .63], *p* < .0001	*b* = 0.66 (0.09), 95% CI [.48, .83], *p* < .0001	*b* = 0.43 (0.09), 95% CI [.26, .61], *p* < .001
**H1: stronger inversion for male targets**			
Target orientation x target sex interaction	*b* = -0.26 (0.13), 95% CI [-.52, -.01], *p* = .045	*b* = 0.21 (0.18), 95% CI [-.13, .56], *p* = .22	*b* = 0.32 (0.18), 95% CI [-.04, .67], *p* = .08
Main effect of inversion: male targets	*b* = 0.60 (0.08), 95% CI [.43, .77], *p* < .0001	*b* = 0.55 (0.12), 95% CI [.32, .79], *p* < .0001	*b* = 0.27 (0.13), 95% CI [.01, .52], *p* = .04
Main effect of inversion: female targets	*b* = 0.35 (0.10), 95% CI [.16, .55], *p* < .001	*b* = 0.77 (0.13), 95% CI [.52, 1.02], *p* < .0001	*b* = 0.58 (0.12), 95% CI [.33, .82], *p* < .001
Target orientation x target sex interaction (covariate: asymmetry)	*b* = -0.26 (0.13), 95% CI [-.52, -.003], *p* = .047	*b* = 0.21 (0.18), 95% CI [-.13, .56], *p* = .22	*b* = 0.32 (0.18), 95% CI [-.04, .67], *p* = .08
**H1b: participants’ gender does not moderate the Target orientation x the target sex interaction**			
Target orientation x target sex x participant gender interaction	*b* = 0.35 (0.26), 95% CI [-.16, .86], *p* = .18	*b* = -0.26 (0.35), 95% CI [-.95, .43], *p* = .46	*b* = 0.09 (0.36), 95% CI [-.62, .80], *p* = .80

**Table 4:** Results of the hypotheses testing for moderation by target variables (standard deviations in parentheses). All studies.

Hypotheses	Study 1	Study 2a	Study 2b
**H2 –lower inversion for more asymmetrical stimuli**			
Target orientation x asymmetry interaction	*b* = -0.07 (0.07), 95% CI [-.21, .07], *p* = .32	*b* = 0.23 (0.10), 95% CI [.03, .42], *p* = .023	*b* = 0.08 (0.10), 95% CI [-.11, .28], *p* = .41
**H3: lower inversion for more sexualized photos**			
Target orientation x sexualization interaction	*b* = -0.04 (0.06), 95% CI [-.16, .09], *p* = .57	*b* = 0.15 (0.10), 95% CI [-.04, .34], *p* = .13	*b* = -0.02 (0.10), 95% CI [-.21, .17], *p* = .84
Target orientation x sexualization x target sex interaction	*b* = -0.11 (0.13), 95% CI [-.36, .16], *p* = .43	*b* = 0.25 (0.30), 95% CI [-.34, .84], *p* = .41	*b* = 0.07 (0.25), 95% CI [-.42, .57], *p* = .77
**H4: higher inversion for more attractive photos**			
Target orientation x attractiveness interaction	*b* = -0.07 (0.06), 95% CI [-.20, .06], *p* = .28	*b* = 0.14 (0.08), 95% CI [-.03, .31], *p* = .10	*b* = -0.08 (0.09), 95% CI [-.26, .10], *p* = .39
Target orientation x attractiveness x target sex interaction	*b* = 0.35 (0.14), 95% CI [.08, .62], *p* = .011	*b* = 0.08 (0.18), 95% CI [-.27, .44], *p* = .65	*b* = 0.14 (0.19), 95% CI [-.23, .51], *p* = .46

**Table 5:** Results of the hypotheses testing for moderation by perceiver variables (standard deviations in parentheses). All studies.

Hypotheses	Study 1	Study 2a	Study 2b
**H5: lower inversion with higher SC-IAT scores**			
Target orientation x SC-IAT interaction	*b* = -0.06 (0.07), 95% CI [-.19, .08], *p* = .40	*b* = 0.01 (0.09), 95% CI [-.17, .18], *p* = .94	*b* = 0.09 (0.09), 95% CI [-.08, .26], *p* = .32
Target orientation x SC-IAT x target sex interaction	*b* = -0.06 (0.14), 95% CI [-.33, .21], *p* = .66	*b* = 0.21 (0.18), 95% CI [-.13, .56], *p* = .23	*b* = 0.11 (0.18), 95% CI [-.24, .46], *p* = .53
**H6: higher inversion for high self-objectifying participants**			
Target orientation x BSH interaction	*b* = 0.02 (0.06), 95% CI [-.10, .14], *p* = .76	*b* = -0.02 (0.09), 95% CI [-.19, .15], *p* = .83	*b* = -0.02 (0.09), 95% CI [-.19, .15], *p* = .81
Target orientation x BSH x target sex interaction	*b* = 0.22 (0.12), 95% CI [-.02, .47], *p* = .07	*b* = -0.26 (0.17), 95% CI [-.60, .08], *p* = .13	*b* = 0.21 (0.17), 95% CI [-.14, .55], *p* = .24
Target orientation x BSV interaction	*b* = 0.06 (0.07), 95% CI [-.07, .19], *p* = .37	*b* = -0.01 (0.09), 95% CI [-.19, .16], *p* = .90	*b* = 0.16 (0.09), 95% CI [-.02, .33], *p* = .08
Target orientation x BSV x target sex interaction	*b* = 0.03 (0.13), 95% CI [-.24, .29], *p* = .84	*b* = -0.32 (0.18), 95% CI [-.67, .04], *p* = .08	*b* = 0.18 (0.18), 95% CI [-.17, .53], *p* = .32
**H7a –effects of benevolent sexism (BS)**			
Target orientation x BS interaction	*b* = -0.03 (0.06), 95% CI [-.16, .10], *p* = .63	*b* = -0.05 (0.09), 95% CI [-.22 .11], *p* = .54	*b* = 0.01 (0.09), 95% CI [-.17, .19], *p* = .93
Target orientation x BS x target sex interaction	*b* = 0.13 (0.13), 95% CI [-.12, .39], *p* = .30	*b* = -0.15 (0.17), 95% CI [-.49, .19], *p* = .38	*b* = -0.06 (0.18), 95% CI [-.43, .30], *p* = .73
**H7b –effects of hostile sexism (HS)**			
Target orientation x HS interaction	*b* = -0.03 (0.06), 95% CI [-.15, .09], *p* = .60	*b* = -0.01 (0.09), 95% CI [-.18, .16], *p* = .94	*b* = -0.07 (0.09), 95% CI [-.24, .11], *p* = .45
Target orientation x HS x target sex interaction	*b* = 0.04 (0.12), 95% CI [-.20, .27], *p* = .77	*b* = -0.04 (0.17), 95% CI [-.38, .30], *p* = .81	*b* = 0.23 (0.18), 95% CI [-.12, .58], *p* = .20
**H7c –effects of benevolent attitude toward men (BM)**			
Target orientation x BM interaction	*b* = 0.03 (0.06), 95% CI [-.09, .16], *p* = .59	*b* = 0.03 (0.09), 95% CI [-.14, .20], *p* = .72	*b* = 0.09 (0.09), 95% CI [-.09, .27], *p* = .34
Target orientation x BM x target sex interaction	*b* = 0.05 (0.13), 95% CI [-.20, .30], *p* = .68	*b* = -0.06 (0.17), 95% CI [-.40, .28], *p* = .73	*b* = -0.01 (0.18), 95% CI [-.37, .35], *p* = .96
**H7d –effects of hostile attitude toward men (HM)**			
Target orientation x HM interaction	*b* = -0.03 (0.06), 95% CI [-.16, .10], *p* = .64	*b* = -0.01 (0.09), 95% CI [-.18, .16], *p* = .93	*b* = -0.04 (0.09), 95% CI [-.23, .14], *p* = .62
Target orientation x HM x target sex interaction	*b* = 0.10 (0.13), 95% CI [-.15, .35], *p* = .44	*b* = -0.12 (0.17), 95% CI [-.47, .21], *p* = .47	*b* = -0.01 (0.18), 95% CI [-.37, .36], *p* = .98

SC-IAT = Single Category Implicit Association Test; BSH = body shame; BSV = body surveillance.

**Table 6:** Results of inversion effects on buildings (standard deviations in parentheses). All Studies.

Hypotheses	Study 1	Study 2a	Study 2b
**Q1 –Inversion effect for buildings**			
Effect of Target orientation	*b* = 0.15 (.09), C.I. [-.02, .33], *p* = .09	*b* = 0.01 (0.09), C.I. [-.17, .18], *p* = .93	*b* = 0.22 (0.09), C.I. [.04, .39], *p* = .016
**Q2 –Effect of asymmetry on inversion (buildings)**			
Asymmetry x Target orientation interaction	*b* = -0.08 (.09), C.I. [-.26, .10], *p* = .39	*b* = -0.17 (0.10), C.I. [-.36, .03], *p* = .09	*b* = 0.02 (0.09), C.I. [-.16, .20], *p* = .80

#### Data analysis

**Overall inversion effect and differences between male and female targets (Table 2):** An overall inversion effect indicated higher accuracy for upright targets. In line with H1, a significant target orientation * target sex interaction indicated a stronger inversion effect for male than female targets, and this interaction was not further moderated by participant gender (H1b). To better understand and quantify the strength of the evidence in favor of the replication of this difference between the inversion effect for male and female targets initially observed by Bernard and colleagues [4], we computed the Bayes Factor (BF), using Dienes calculator (www.lifesci.sussex.ac.uk/home/Zoltan_Dienes/inference/bayes_factor.swf). Following Dienes [73], we compared the null hypothesis of no difference in the inversion effect between male and female targets with the alternative hypothesis of the presence of a stronger inversion effect for male targets with size b = .24 (this size was estimated based on the difference between the inversion effect for male and female participants in [4]) and SD = .12. The analysis yielded BF10 = 5.39, which is interpreted as moderate evidence [74] in favor of a successful replication.

**Target variables (Table 4):** The target orientation * asymmetry interaction had a negative sign, indicating that in our sample lower asymmetry was associated with stronger inversion effects. This pattern was consistent with H2 but, importantly, this effect was not significant. Nevertheless, to check the alternative explanation according to which the difference in inversion effects between male and female targets might be due to differences in asymmetry, we performed a second test of H1, entering asymmetry as a covariate. However, when asymmetry was statistically controlled, target sex still moderated the inversion effect, ruling out the alternative explanation.

Contrary to H3, the target orientation * target sexualization interaction was not significant: No evidence emerged that sexualization influenced the inversion effect. One could argue that sexualization, perhaps, specifically influences the objectification of women; however, the target orientation * target sexualization * target sex interaction was not significant, providing no support for this additional hypothesis.

For attractiveness (H4), a significant target orientation * target attractiveness * target sex interaction emerged. To understand this interaction, we explored the orientation * target attractiveness interaction, separately for male and female targets. This analysis showed that attractiveness significantly moderated the inversion effect for male targets, *b* = - 0.18, *SE =* .09, *p* = .04: Consistent with H4, pictures of male targets considered as more attractive by the participants were associated with a smaller inversion effect. For female targets, however, the interaction effect was not significant and, if anything, in the opposite direction, *b* = 0.16, *SE =* .10, *p* = .11.

**Perceiver variables (Table 5):** Contrary to H5, the woman-human SC-IAT score did not moderate the inversion effect. Both the target orientation * SC-IAT and the target orientation * SC-IAT * target sex interactions failed to reach statistical significance.

To investigate whether self-objectification moderated the inversion effect, we conducted separate analyses for the BSH and BSV scores. Contrary to H6, neither the target orientation * BSH, nor the target orientation * BSV interactions were significant.

Contrary to H7, the inversion effect was not moderated by any of the four subscale scores.

**Buildings (Table 6):** Target orientation had no significant impact on the accuracy of recognition of buildings (Q1), and the target orientation * asymmetry interaction was not significant (Q2).

### Discussion

Using the same stimulus materials of Bernard and colleagues [4], we replicated the stronger inversion effects for sexualized men as compared to women (H1), independently from the gender of participants (H1b). The inversion effect was present for both female and male targets, while it did not emerge in accuracy data for objects (Q1). Asymmetry did not moderate the inversion effect. Therefore, the lower inversion effect for female targets observed here and in the original studies of Bernard and colleagues [4, 5] cannot be easily dismissed as an artifact caused by the higher asymmetry of female targets. Only one of the variables we investigated for construct validity was significantly related to the size of the inversion effect: attractiveness. However, the results on attractiveness only partially supported H4, and therefore the validity of the IBRT as a measure of objectification. Indeed, based on the existing literature we expected objectification to be positively related to attractiveness of the targets, leading to a decrease in the inversion effect. In the present study, we found that attractiveness of male targets was indeed associated with lower levels of objectification, but this same pattern did not emerge for female targets.

We checked whether the lack for more substantive evidence of validity of the IBRT is due to an insufficient level of power in our analyses. To tackle this concern, first, it is worth noting that random effect models, such as those we used in the present analysis, substantially increase the power of the design by decreasing the standard error of the coefficient estimates [75] and are, therefore, more powerful than the fixed effect models that so far have been used—with comparable or lower sample sizes—to investigate the IBRT. Furthermore, using the *simr* package [76] in R, we ran Monte Carlo simulations to estimate the sensitivity of our research design. With 400 simulations for each of the effects, α = .05, and (1-β) = .80, this simulation estimated that our empirical design had a minimal detectable effect, computed as Cohen’s *d*, of *MDE* = 0.10, and influences on this effect by our moderators had a *MDE =* 0.12. Based on Cohen [77], these values indicate that our design reached the conventional level of power of (1-β) = .80 even for inversion and moderation effects of small size. As concerns the three-way interactions between target orientation, target sex, and the moderators, the *MDE* equaled 0.28. Finally, as we might expect a specific impact of the moderators on the inversion effect for female targets, we checked the sensitivity of the target orientation * moderator interaction, when restricted to female targets. Simulations indicated that our design reached the conventional level of power of (1-β) = .80 for *MDE* = 0.16. In sum, this sensitivity analysis (the details of which are available in the S4 File) suggests that the lack of evidence for the construct validity of IBRT is not attributable to insufficient power of the research design.

In the next step of the research, we tested the hypotheses with different materials. This not only allowed to check whether H1 would be confirmed with other stimuli, but also provided a safeguard against Type-I errors for the other hypotheses that we examined to thoroughly investigate the construct validity of the IBRT.

### Study 2a

Study 2a was identical to Study 1, with the exceptions that different sexualized male and female photos were presented to participants in the IBRT (see previous section Materials, New Stimuli) and that male and female targets had similar levels of asymmetry.

### Data cleaning

One participant had outlying performance in the Inverted Building Recognition Task, and one had a bad performance (below 75% correct) on the SC-IAT; hence, their responses on these tasks were treated as pairwise missing.

A synopsis of the results is presented in Tables 3–6, center column, and S3 File contains the complete analyses on both accuracy and latency.

### Data analysis

**Overall inversion effect and differences between male and female targets:** Participants answered more accurately to right-side up than upside-down stimuli, but the target orientation * target sex interaction was not significant: H1 was not replicated. If anything, the effect of target sex was in the opposite direction, indicating stronger effects of target orientation on accuracy for female than male targets. As we had done for Study 1, we computed the BF to compare the evidence against (null hypothesis) and supporting the replication of the results found by Bernard and colleagues [4]. The analysis yielded BF01 = 5.26, which is interpreted as moderate evidence in favor of the null hypothesis.

Finally, the three-way interaction among participant gender, target sex, and target orientation (H1b) was not significant, indicating that male and female participants showed the same pattern of results.

**Target variables:** Unlike Study 1, a significant target orientation * asymmetry interaction emerged. Contrary to H2, however, the sign of the interaction was positive, indicating that higher asymmetry was associated to an increase in inversion. Contrary to H3, target sexualization did not moderate either the effect of target orientation or the target orientation * target sex interaction.

Contrary to H4, target attractiveness did not interact with target orientation, and it did not moderate the target orientation * target sex interaction.

**Perceiver variables:** Replicating Study 1, the woman-human SC-IAT (H5), self-objectification (H6) and sexism (H7) did not moderate the inversion effect.

**Buildings:** As in Study 1, no inversion effect was observed for building (Q1). Also, the asymmetry * target orientation interaction failed to reach significance (Q2).

### Discussion

The use of different sexualized stimuli in an otherwise unaltered experimental setting led to very different results. In Study 2a we found a significant and strong inversion effect for the new set of sexualized stimuli. The size of this effect is approximately the same as in our Study 1. However, the inversion effect is not significantly different for photos of male and female targets and, if anything, it suggests a stronger inversion effect for female targets. Therefore, the main result from the studies of Bernard and colleagues [4, 5], and of our Study 1, is not replicated. The main claim of Bernard and colleagues that female sexualized stimuli suffer lower inversion effects and the subsequent deduction that they are more objectified than men are not supported using different materials. Stimulus asymmetry impacts the inversion effect, albeit in an unexpected way: The higher the asymmetry, the higher the inversion effect. These two results, taken together, underline the importance of specific characteristics of the stimuli in the inversion effect. Finally, none of the other target and perceiver variables impacted our dependent variable.

As for Study 1, we conducted a Monte Carlo simulation, to check if our design was adequately powered to capture the effects of interest. The overall number of female and male stimuli presented to participants in Study 2a was half as much as in Study 1, which led to a small decrease in the overall sensitivity of the design. However, also for Study 2a the sensitivity analysis, conducted with 400 simulations for each effect, α = .05 and (1-β) = .80, showed that our design could capture the inversion effect with *d* ≥ 0.13, moderations of this effect by sex of target with *d* ≥ 0.27, and by the other moderators with *d* ≥ 0.15. For the three-way interactions between target orientation, target sex, and each of the moderators, the design was sufficiently powered to capture interaction effects with *d* ≥ 0.33 (with the exception of the target orientation * target sexualization * target sex interaction, where *MDE* = 0.50). For analyses restricted to female targets, the design had power (1-β) = .80 to capture target orientation * moderator interactions with *MDE* = 0.24. In sum, even though the number of images used in Study 2a was half of the number used in Study 1, the experiment had power (1-β) = .80 to capture effects with small-to-low size (the details of the sensitivity analysis are available in the S4 File).

Following the request of a reviewer, we further conducted a meta-analytic summary of the moderation effects that emerged in Study 1 and Study 2a. This analysis aimed at checking the robustness of the results, and is reported in detail in the S5 File. When the data of the two studies using sexualized targets were pooled together, the Bayesian analysis yielded a Credibility Interval for the difference between the inversion effect of male and female participants of 95%Cr = [-0.30, 0.11], with BF01 = 1.41, which is considered as inconclusive, anecdotal evidence in favor of the null hypothesis. This confirmed that the presence or absence of a difference in the inversion effect for male and female targets was contingent on the specific dataset used: Study 1, with the original dataset of Bernard and colleagues [4], yielded support for the presence of this difference, while Study 2a, with a newly created dataset of controlled sexualized stimuli, provided support for its absence. Finally, the aggregation of results from the two studies (which were based on samples of participants from the same population, collected in the same laboratories, in very similar conditions) provided anecdotal evidence for the null hypothesis. The aggregated analysis also confirmed that male and female participants showed the same pattern of results (H1b confirmed). Furthermore, none of the interactions involving the moderators was confirmed on the aggregated data.

### Study 2b

#### Data cleaning

Three participants had outlying performance in the Inverted Building Recognition Task, and two participants had a performance below 75% correct on the SC-IAT; hence, these data were pairwise deleted.

A synopsis of the results is presented in Tables 3–6, right column, and, as for previous studies, a complete report of all accuracy and latency results is reported in S3 File.

#### Data analysis

**Overall inversion effect and differences between male and female targets:** The inversion effect was significant, showing higher accuracy for upright stimuli. No significant difference emerged in the inversion effect for male and female targets (H1). If anything, the effect of target orientation was stronger for female as compared to male targets. Also the interaction among participant gender, target sex and target orientation was not significant (H1b).

**Target variables:** As in Study 1, and inconsistent with H2, analyses showed no significant interaction between asymmetry and target orientation. Similarly, neither sexualization (H3) nor attractiveness (H4) of the stimuli moderated the inversion effect.

**Perceiver variables:** No significant effect emerged for the woman-human SC-IAT (H5), self-objectification (H6), and gender attitudes (H7).

**Buildings:** Differently from the previous studies, the inversion effect for buildings was significant (Q1). Again, however, no asymmetry * target inversion effect was observed (Q2).

### Discussion

In the present study, using photographs of non-sexualized women and men, we found the overall inversion effect for human stimuli, with no difference between female and male targets and no effect of the asymmetry. Different from Study 1 and Study 2a, the inversion effect emerged also for buildings. This difference from the previous studies was unexpected because we used the same pictures of buildings in all studies. This shows that inversion effects are highly volatile: Their presence and size are related not only to the specific characteristics of the stimuli but also to unknown characteristics of the administration setting.

None of the other variables investigated to provide construct validity to the IBRT as a measure of objectification significantly interacted with the inversion effect.

## General discussion

As outlined in the introduction, the inversion effect, or impaired performance in the recognition of upside-down as compared to upright stimuli, has been proposed by Bernard and his colleagues [4] as an indicator of human-like versus object-like perception, which could prove that, under certain circumstances, humans can be processed as objects. Based on the observation that male sexualized photos suffered stronger inversion effects than female ones, the authors concluded that sexualized women are perceived as objects. However, the inversion effect should be considered at best as an indirect measure of human-like perception, which captures whether stimuli undergo configural versus analytical processing. The interpretation of indirect measures such as this one is complex because various characteristics of the stimuli can influence performance, especially when these are complex photographs of real individuals (the variability is more easily controlled when simpler body silhouettes are used as stimuli). Therefore, great caution and extensive validation should accompany the use of such a measure.

The recent replication crisis in psychology, and in other fields of science, has highlighted the importance of independent replications of results within new laboratories (e.g., [78–80]). Given the potential usefulness of the task developed by Bernard and colleagues [4], we deemed it important to better investigate it through replication.

As a first step, we checked and ruled out the role of asymmetry as a confounding variable causing the differences between inversion effects for male and female targets [27, 28]: In Study 1, in which female targets were significantly more asymmetrical than their male counterparts, the difference in the size of the inversion effect was still present. Moreover, the level of asymmetry of the stimuli did not moderate the inversion effect in Study 1 and Study 2b. In Study 2a, contrary to the concern that higher asymmetry may cause lower inversion effects [27, 28], it was associated with higher inversion effects. Furthermore, asymmetry never moderated the inversion effect for objects. Finally, to check the possibility that asymmetry in the areas of secondary sexual zones might play a role, we computed an index of asymmetry of the bust area. Even the analyses performed on this index (reported in S5 File) confirmed the results obtained with the general asymmetry index.

**Fig 2:**
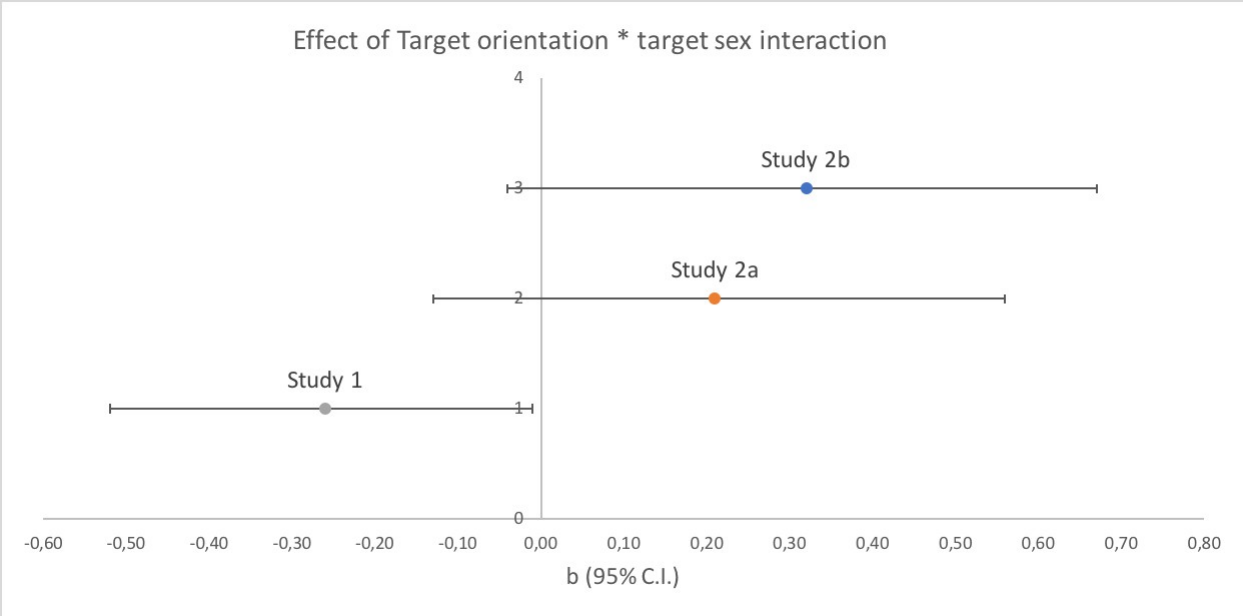
Effect of Target orientation * target sex interaction across studies, with 95% confidence intervals.

The next step consisted in investigating whether the inversion effect was related to the humanization vs. objectification of the stimuli. In all studies, we found strong and reliable inversion effects for human photos. Only in Study 2b, did a significant inversion effect also emerge for high-rise buildings—albeit it was smaller than for human stimuli. This inversion effect for buildings is consistent with the literature [28, 32, 38–40] and it indicates that inversion *per se* is insufficient to signal that the stimulus is processed as a human being. In light of this, a proponent of the use of the IBRT as measure of objectification could argue that the important aspect is not the presence versus absence of the inversion effect, but its intensity: Less inversion effect would be associated with objectified stimuli, stronger inversion effect with humanized stimuli. From a theoretical point of view, the presence of differences in the inversion effect between male and female sexualized targets is important, because based on objectification theory [7] we expect that sexualized women are objectified more strongly, as compared to sexualized men. However, we found a stronger inversion effects for sexualized male as compared to female photographs only with the original set of stimuli (Study 1), but not with other material (Studies 2a and 2b); Fig 2 shows a summarizing graph comparing the interaction effect for H1 across studies with corresponding 95% confidence intervals. This confirmed that the inversion effect by target sex is highly replicable (in the direct sense, i.e., with the original stimuli) but not at all generalizable. It is important to underline that Study 1 and Study 2a were very similar: Participants were sampled from the same population and tested in the same laboratory, with the same experimental procedure and materials, the only substantial difference being the stimuli used (which had undergone pretest for various variables, including asymmetry and sexualization, see S1 File). Therefore, the absence of differences in the inversion effect for male and female targets in Study 2a cannot be easily dismissed as related to circumstantial aspects, as the very same paradigm produced the difference under scrutiny in Study 1. To the best of our knowledge, this is the first research that provides such a stringent test for the role of the specific stimuli used in the IBRT.

Based on the rationale that, perhaps, the *size* of the inversion effect is an indicator of objectification, in the three studies, we put the IBRT score in relation to many variables that were expected to affect objectification.

None of the hypotheses we put forward to test the IBRT construct validity was clearly supported. A particularly problematic result concerns the absence of any impact of sexualization on the inversion effect. Sexualization and sexual objectification are conceptually distinct constructs that are expected to be related from the theoretical point of view and studies conducted with other measures of objectification provide empirical evidence that this is the case [51]. However, in none of the studies emerged any support for a relationship between sexualization and the inversion effect at the level of the individual stimuli. Furthermore, an inspection of Table 2 shows that the difference in accuracy between upright and upside-down human stimuli is approximately the same in the three studies (this difference is .05 in Study1, .08 in Study 2a, and .05 in Study 2b), despite the fact that the stimuli were sexualized in Study 1 and Study 2a, but not in Study 2b.

Also the results emerging from the investigation of the link between the inversion effect and various moderators, chosen on the basis of theory and previous empirical evidence, did not provide results supporting the construct validity of the IBRT, although the sensitivity analysis indicates that our studies were able to capture moderation effects of small-to-medium size: No evidence emerged that targets’ sexualization, perceivers’ automatic woman-human association, self-objectification or gender attitudes moderated the inversion effect.

As regards the attractiveness of the target, which was related to an increase in objectification in previous empirical evidence [54], we found one piece of evidence that supported previous results. Namely, in Study 1 the higher was the attractiveness of the male targets, the lower was the inversion effect that emerged. However, this effect did not emerge for female targets, was not replicated in the following studies, nor supported by the aggregated analysis of Study 1 and Study 2a (S6 File): therefore, the most reasonable conclusion from the overall pattern of results is that it should be treated as a case of Type 1 error.

In sum, this overall pattern of results indicates that great caution should be used in interpreting the inversion effect in IBRT as an indicator of objectification.

The present results confirm the strong impact of the specific stimuli on the inversion effect. Given this high contingency of results on the specific stimuli, any difference between conditions in which different stimuli are used is uninformative. It is worth noticing that our sets of stimuli were not as controlled as those generally used in perception studies. However, as noted and contrary to the original set of stimuli used by Bernard and colleagues [4], our images were controlled for symmetry, level of sexualization, and the same male and female models were used in both Studies 2a and 2b. As the stimuli from Study 2a were rated as significantly less sexualized than the stimuli from Study 1, we might wonder if this could be part of the reason underlying the failed conceptual replication. However, this seems implausible for two reasons: First, as we mentioned, all stimuli in both studies were highly sexualized, falling within the “sexualized” or “hypersexualized” categories, as defined by Hatton and Trautner [51, 52]. In other words, each image contained various different attributes related to sexualization (sexualized pictures), or a combination of so many sexualized attributes that the possible interpretation of the image was narrowed to the sex (hypersexualized pictures). Second, and most importantly, in none of the studies did sexualization moderate the inversion effect.

It is important to stress that we found differences in the inversion effects when we used the same images of buildings: The inversion effect was significant in Study 2b, nonsignificant but still in the same direction in Study 1, and virtually absent in Study 2a. This indicates that even with the same non-human stimuli, the specific conditions may strongly impact the results, further speaking against using the IBRT to infer human-like or object-like perception of stimuli.

Notably, we were able to find these results due to the application of mixed-effects models with crossed random effects. The latter are particularly adequate to the present context, given that the specific stimuli are a random selection of the universe of the possible stimuli. In fact, fixed effects modeling assumes homoscedasticity and does not allow an adequate analysis of interactions between categorical and continuous covariates [81]. Discarding simultaneous items and subjects on variability, as in fixed effects model assumptions, yielded less-trustworthy results.

## Conclusions

The present results do not support the use of the IBRT as a measure of sexual objectification. The inversion effects were unrelated to any of the social variables (sex, sexualization and attractiveness of the stimuli; self-objectification, sexism and automatic associations of the perceiver) in a convincing way. Moreover, the results were clearly unstable. The inversion effect observed in the IBRT is probably highly dependent on the specific set of stimuli and on other, so-far unknown, aspects of the administration conditions. Taken together, in line with perceptual research on BIE [33–35], these results call for more research to understand which are the aspects of human stimuli that most strongly impact inversion, and they suggest that high methodological attention should be paid in designing research using the IBRT (for instance, one should always use at least two different stimulus sets to ensure that the results are not due to idiosyncratic characteristics of materials).

Even if we were to put aside for a moment this overall pattern of absence of moderation effects and accept the body inversion effect as a measure of configural processing, and hence as an indirect indication of human-like (as opposite to object-like) processing of stimuli, our data speak against considering the results reported by Bernard and colleagues [4, 5] as proof that sexualized women are perceived in an object-like way. Indeed, our Study 1 shows that using the same stimuli as Bernard and colleagues, the inversion effect for female targets is significant and much greater in size as compared to the inversion effect for objects. Importantly, it is sufficient to use different sexualized stimuli, as we did in Study 2a, and the difference in inversion between male and female targets completely disappears.

To conclude, it is important to stress that the present research specifically investigated one possible indicator of objectification, namely the decreased inversion effect in the IBRT, and shows that this indicator is highly volatile. However, this should not be interpreted as an indication that objectification *per se* is a volatile phenomenon. Quite to the contrary, many recent studies have shown the prominence of sexual objectification in cognitive processing [6], and in daily life [82]. Therefore, it is crucial to continue studying this phenomenon, but with different methods and measures. For instance, a promising way to assess sexual objectification comes from studies investigating it at the neurophysiological level (e.g., [48, 83, 84]). In particular, empirical research has pointed to the N170, which is a negative amplitude Event Related Potential evoked by visual stimuli at occipitotemporal regions, approximately 140 to 200 ms after stimulus onset. Empirical evidence indicates that the N170 might be important for the investigation of configural processing of human bodies, as this neurophysiological response is stronger for bodies and faces than for objects (e.g., [33, 85, 86]). Moreover, the amplitude of N170 has been shown to be increased for images of bodies presented upside down, as compared to upright but, interestingly, this increase in amplitude did not emerge for objects [86]. Recently, Bernard and colleagues [48, 83, 84] showed that non-sexualized bodies presented larger N170 amplitudes when viewed upside-down than in upright positions, whereas bodies with sexually suggestive posturer failed to evoke a stronger N170 response when presented upside-down. This result suggests that the amplitude of the inversion in N170 might be a useful signal that stimuli are processed configurally. However, this happened both for images of male and female bodies indicating that, if this result is related to cognitive objectification, no difference emerged in the objectification of male and female targets.

This line of research could be useful for the investigation of objectification. However, because it is just at its beginning, results need to be replicated with different stimuli, and in different research laboratories (see [87]). This would also require a thorough investigation of whether the effect on the N170 amplification for inverted sexualized stimuli is an effective indicator of cognitive objectification. In other words, it would be important to verify whether a lower increase in N170 in response to certain stimuli can be univocally traced back to a lower ascription of humanity, because we cannot exclude that other processes, besides cognitive objectification, might be at play (see [33]).

Precisely because objectification is a tangible and important phenomenon, which impact on the well-being of many people, it is important to investigate it with effective and valid instruments. It is also important to notice that our results, and in particular the absence of a conceptual replication for the result of higher inversion effects for male as compared to female targets, should not be considered as evidence that the sexualized-body-inversion hypothesis [4] is disconfirmed. The present findings more specifically show that the IBRT, as an instrument, presents validity issues, and, as a consequence, using it to test the sexualized-body-inversion hypothesis might produce misleading conclusions.

## Supporting information

S1 File Developing a new set of stimuli. (DOCX)

S2 File Results for self-objectification (OBCS) and gender beliefs (ASI, AMI). (DOCX)

S3 File Detailed results. (DOCX)

S4 File Sensitivity analysis. (DOCX)

S5 File Bust area asymmetry. (DOCX)

S6 File Ancillary Analysis: Bayesian meta-analytical aggregation of the results of Study 1 and Study 2a. (DOCX)
