# Supplementary material for: Measuring objectification through the Body Inversion Paradigm: Methodological issues
Source: PLoS One. 2020 Feb 19;15(2):e0229161. doi: 10.1371/journal.pone.0229161 (PMC7031944; doi:10.1371/journal.pone.0229161)
Supplement: S1 File — (DOCX) [file pone.0229161.s001.docx]

**Measuring Objectification Through the Body Inversion Paradigm: Methodological Issues**

*Cristina Zogmaister, Federica Durante, Silvia Mari, Franca Crippa, & Chiara Volpato*

*University of Milano - Bicocca*

**Supplementary Online Materials**

**S1. Developing a new set of stimuli**

The aim of this pretest was to create a new set of images depicting men and women with similar postural asymmetry.

*Material*

We retrieved the photographs in March 2014 from different websites. We initially searched for photos that portrayed the same models fully dressed and wearing bathing suits or underwear (undressed, hereafter) in an upright position against a white background. Our initial set comprised 112 images. The individuals were portrayed horizontally centered and had mostly neutral facial expressions. The size of the images was rescaled to 500 * 750 pixels and the clothing brands were removed.

We computed the postural asymmetry of each picture based on eight points of the bodies: chin, navel, top of the head, elbows, shoulders, hips, hands, and legs. For unique points (chin, navel, forehead), we considered the Cartesian distance from the vertical line in the center of the image, whereas for double points (elbows, shoulders, hips, hands, legs), we considered the Cartesian distance between the right point and the mirror image of the left point.

For each photograph, two independent raters measured the following points in pixels:

x1: top of the head;

x2: chin;

x3: navel;

(x4_a_, y4_a_): left elbow;

(x4_b_, y4_b_): right elbow;

(x5_a_, y5_a_): left shoulder;

(x5_b_, y5_b_): right shoulder;

(x6_a_, y6_a_): left hip;

(x6_b_, y6_b_): right hip;

(x7_a_, y7_a_): left hand, end of middle fingertip;

(x7_b_, y7_b_): right hand, end of middle fingertip;

(x8_a_, y8_a_): external part of the left leg, where it touches the border of the image;

(x8_b_, y8_b_): external part of the right leg, where it touches the border of the image.

Based on these points:

1. we computed the X coordinate of the center of the image;
2. for the three unique points, we computed the values dist1, dist2, and dist3 as distances between the measured points and the center;
3. for the five double points, we computed the X coordinate of the mirror image, mirrored on the center as computed in (a);
4. for the five double points, we computed the values dist4, dist5, dist6, dist7, and dist8; each was the distance between the coordinates (x_a_, y_a_) and (x_b_specular, y_b_);
5. we computed the sum of all distances dist1, dist2, dist3, dist4, dist5, dist6, dist7, and dist8.

Therefore, for each image and for the measurements provided by each judge, we obtained a single value (i.e., Asymmetry Body Posture Index; ABPI) that summarized the overall degree of postural asymmetry. Higher values correspond to higher levels of asymmetry.

Please find the SPSS syntax for the computation of asymmetry hereafter:

| *** SPSS syntax for the computation of the asymmetry index  *** Measuring Objectification Through the Body Inversion Paradigm: Methodological Issues  ** computation of the vertical X axis - the center of the image **  COMPUTE Xcenter = mean (X1,X2,X3, mean(X4a, X4b), mean(X5a, X5b), mean(X6a, X6b), mean(X7a, X7b), mean(X8a, X8b)).  EXECUTE.  ** computation of the distance between the unique points and the center of the image **  COMPUTE dist1=abs(X1-Xcenter).  COMPUTE dist2=abs(X2-Xcenter).  COMPUTE dist3=abs(X3-Xcenter).  ** computation of the specular points **  compute x4b_s = Xcenter * 2 - x4b.  compute x5b_s = Xcenter * 2 - x5b.  compute x6b_s = Xcenter * 2 - x6b.  compute x7b_s = Xcenter * 2 - x7b.  compute x8b_s = Xcenter * 2 - x8b.  ** computation of cartesian distances **  compute dist4=sqrt((x4a-x4b_s)*( x4a-x4b_s)+(y4a-y4b)*(y4a-y4b)).  compute dist5=sqrt((x5a-x5b_s)*( x5a-x5b_s)+(y5a-y5b)*(y5a-y5b)).  compute dist6=sqrt((x6a-x6b_s)*( x6a-x6b_s)+(y6a-y6b)*(y6a-y6b)).  compute dist7=sqrt((x7a-x7b_s)*( x7a-x7b_s)+(y7a-y7b)*(y7a-y7b)).  compute dist8=sqrt((x8a-x8b_s)*( x8a-x8b_s)+(y8a-y8b)*(y8a-y8b)).  EXECUTE.  ** computation of asymmetry as sum of the indicators  COMPUTE asymmetry=sum(dist1, dist2,dist3,dist4,dist5,dist6,dist7,dist8).  EXECUTE. |
| --- |

The inter-rater reliability for ABPI was excellent, *r* = .94 (α = .97). The asymmetry scores from the two judges were averaged, and the mean was used in the following analyses.

We selected two photos for each of 12 female and 12 male models, one that portrayed the model dressed and one undressed, for a total of 48 different pictures characterized by comparable levels of asymmetry.
An ANOVA with ABPI as the dependent variable and target gender and clothing (dressed, undressed) as a between-participants factor was run, and the results showed no significant results, *F*s < 1. In other words, no significant difference in terms of postural asymmetry emerged depending on either the sex or clothing of the models.

This new set of stimuli was then pre-tested.

*Participants*

Forty-one participants (90.3% university students), 21 female, *M*_age_ = 22.98, *SD* = 2.59, took part in the pre-test in exchange for course credits.

*Procedure*

The pre-test was run via computer. Participants were welcomed individually in the laboratory and were asked to sit in front of the computer and read the instructions. The study was presented as a pre-test of a new set of images to use in future experiments on the objectification of women. Then, the 48 images were presented one after the other on the computer screen in random order. For each image, participants were asked to indicate to what extent the model was good-looking, vulgar, attractive and sexually provocative on a 5-point scale, *1* = *not at all*, *3* = *averagely*, *5* = *extremely*.

*Results*

We checked the reliabilities for the evaluations of how good-looking, vulgar, attractive and sexually provocative the models were separately for the images of female and male models. All Cronbach’s alphas were excellent (all α ≥ .91). We averaged the evaluations across participants in order to have an evaluation of each dimension for each of the models, and we subsequently investigated the correlations between these average scores (see Table 1).

Table 1. *Correlations, all images*

|  | | ABPI | Good-Looking | Vulgar | Attractive |  |
| --- | --- | --- | --- | --- | --- | --- |
| Good-looking |  | .264 |  |  |  | |
| Vulgar |  | .215 | .131 |  |  |  |
| Attractive |  | .301^*^ | .965^**^ | .240 |  |  |
| Sexually provocative |  | .307^*^ | .570^**^ | .752^**^ | .692^**^ |  |

^**^ *p ≤* .01; ^*^ *p ≤* .05.

Good-looking was highly correlated with attractive, and sexually provocative was highly correlated with vulgar. Interestingly, good-looking was not correlated with vulgar. Remarkably, the asymmetry index (ABPI) only correlated with how attractive and sexually provocative the targets were perceived.

When we correlated the above variables for dressed and undressed targets separately, we found similar correlations for the dressed targets, but the ABPI marginally correlated with vulgar for the undressed targets (see Table 2 and 3).

Table 2. *Correlations for dressed targets*

|  | | ABPI | Good-Looking | Vulgar | Attractive |
| --- | --- | --- | --- | --- | --- |
| Good-looking |  | .381^†^ |  |  |  |
| Vulgar |  | -.022 | -.083 |  |  |
| Attractive |  | .450^*^ | .979^***^ | -.055 |  |
| Sexually provocative |  | .430^*^ | .493* | .674^***^ | .569^**^ |

^**^ *p ≤* .01; ^*^ *p ≤* .05; ^†^ *p* =.067. *N* = 24

Table 3. *Correlations for undressed targets*

|  | | ABPI | Good-Looking | Vulgar | Attractive |
| --- | --- | --- | --- | --- | --- |
| Good-looking |  | .143 |  |  |  |
| Vulgar |  | .385^†^ | .358^††^ |  |  |
| Attractive |  | .143 | .958^***^ | .465^*^ |  |
| Sexually provocative |  | .228 | .724^***^ | .740^***^ | .834^***^ |

^†^ *p* =.063, ^††^ *p* =.086. *N* = 24

Finally, 2 (Target gender: Male vs. Female) x 2 (Clothing: Dressed vs. Undressed) ANOVAs were performed on the four items, using the index of asymmetry as a covariate.

*Good-looking*: the covariate was significant *F* (1, 43) = 4.48, *p* = .04, *η*^2^*_p_* = .09. Only Target Gender resulted significant, *F* (1, 43) = 10.11, *p* = .003, *η*^2^*_p_* = .19: women were rated more good-looking (*M* = 3.17, *SD* = 0.57) than men (*M* =2.71, *SD* = 0.48).

*Vulgar*: the covariate was not significant, thus it was removed from the model. Results showed only a main effect of Clothing, *F* (1, 44) = 15.06, *p* < .001, *η*^2^*_p_* = .26: targets wearing only underwear or bathing suits were rated more vulgar (*M* = 1.80, *SD* = 0.44) than when they were fully dressed (*M* = 1.39, *SD* = 0.30). [results were almost identical when the covariate was included in the model].

*Attractive*: the covariate was significant *F* (1, 43) = 5.01, *p* = .03, *η*^2^*_p_* = .10. Only Target Gender resulted significant *F* (1, 43) = 5.37, *p* = .025, *η*^2^*_p_* = .11: women were rated more attractive (*M* = 2.94, *SD* = 0.58) than men (*M* = 2.60, *SD* = 0.49).

*Sexually provocative*: the covariate was significant *F* (1, 43) = 4.50, *p* = .04, *η*^2^*_p_* = .10. Results showed only a main effect of Clothing, *F* (1, 43) = 5.00, *p* = .031, *η*^2^*_p_* = .10: targets wearing only underwear or bathing suits were rated more sexually provocative (*M* = 2.44, *SD* = 0.67) than when they were fully dressed (*M* = 2.07, *SD* = 0.40).
