# Supplementary material for: Measuring objectification through the Body Inversion Paradigm: Methodological issues
Source: PLoS One. 2020 Feb 19;15(2):e0229161. doi: 10.1371/journal.pone.0229161 (PMC7031944; doi:10.1371/journal.pone.0229161)
Supplement: S2 File — (DOCX) [file pone.0229161.s002.docx]

**S2. Results for self-objectification (OBCS) and gender beliefs (ASI, AMI)**

**Self-Objectification (OBCS)**

Table 1. *Descriptive analyses and correlations between subscales of OBCS, and test of the differences between female and male participants.*

|  | Scale | *N* | Min | Max | *M* | *SD* | Correlations and reliabilities (reliabilities on the main diagonal) | | | Female  participants | | | | Male participants | |
| --- | --- | --- | --- | --- | --- | --- | --- | --- | --- | --- | --- | --- | --- | --- | --- |
|  |  |  |  |  |  |  | BSV | BHS | | *M* | | *SD* | | *M* | *SD* |
|  |  |  |  |  |  |  |  |  | |  | |  | |  |  |
| Study 1 | BSV | 97 | 1.75 | 5.63 | 4.37 | 0.89 |  |  | | 4.55 | | 0.73 | | 4.18 | 0.99 |
|  | BSH | 97 | 1.25 | 6.00 | 3.34 | 1.08 | .60^***^ |  | | 3.49 | | 1.14 | | 3.19 | 1.00 |
|  | ACB | 96 | 2.75 | 6.75 | 4.92 | 0.77 | .15 | .00 | | 4.72 | | 0.71 | | 5.12 | 0.77 |
|  |  |  |  |  |  |  |  |  | |  | |  | |  |  |
| Study 2a | BSV | 85 | 1.88 | 6.63 | 4.16 | 1.01 |  |  | | 4.34 | | 0.93 | | 3.97 | 1.08 |
|  | BSH | 85 | 1.25 | 6.13 | 3.23 | 1.19 | .56^**^ |  | | 3.49 | | 1.29 | | 2.96 | 1.02 |
|  | ACB | 85 | 3.50 | 7.00 | 5.05 | 0.82 | .12 | .04 | | 4.91 | | 0.60 | | 5.20 | 0.98 |
|  |  |  |  |  |  |  |  |  | |  | |  | |  |  |
| Study 2b | BSV | 92 | 1.00 | 6.50 | 4.13 | 1.10 |  |  | | 4.48 | | 1.00 | | 3.77 | 1.09 |
|  | BSH | 92 | 1.00 | 6.75 | 3.46 | 1.22 | .66** |  | | 3.78 | | 1.31 | | 3.12 | 1.02 |
|  | ACB | 92 | 2.75 | 6.75 | 4.90 | 0.84 | -.31** | -.33** | | 4.73 | | 0.77 | | 5.07 | 0.89 |
|  |  |  |  |  |  |  |  |  |  | |  | |  |  | |

*Note*. ^***^*p* < .001; ^**^*p* < .01; ^*^*p* < .05

In line with the literature (Calogero, 2011; Moradi & Huang, 2008) female participants had significantly or tendentially higher scores on the BSV scale [for Study 1, *t*(95) = 2.06, *p* = .042; for Study 2a, *t*(83) = 1.70, *p* = .093; for Study 2b, *t*(90) = 3.24, *p* = .002], significantly or tendentially lower scores on the ACB scale [for Study 1, *t*(95) = 1.40, *p* = .011; for Study 2a, *t*(83) = 1.63, *p* = .11; for Study 2b, *t*(90) = 1.95, *p* = .054], and significantly or marginally higher scores on the BSH subscale [for Study 1, *t*(95) = 1.40, *p* = .16; for Study 2a, *t*(83) = 2.10, *p* = .039; for Study 2b, *t*(90) = 2.67, *p* = .009].

**Ambivalent Sexism Inventory (ASI) and Attitude Toward Men Inventory (AMI)**

Table 2. *Descriptive analyses and correlations between subscales of ASI and AMI, and test of the differences between female and male participants*.

|  | Scale | *N* | Min | Max | *M* | *SD* | Correlations and reliabilities (reliabilities on the main diagonal) | | | Female participants | | Male participants | | |  |
| --- | --- | --- | --- | --- | --- | --- | --- | --- | --- | --- | --- | --- | --- | --- | --- |
|  |  |  |  |  |  |  | ASI BS | ASI HS | AMI BM | *M* | *SD* | | *M* | *SD* | |
|  |  |  |  |  |  |  |  |  |  |  |  | |  |  | |
| Study 1 | ASI BS | 97 | 1.00 | 6.55 | 3.89 | 1.06 |  |  |  | 3.91 | 1.02 | | 3.86 | 1.11 | |
|  | ASI HS | 97 | 1.18 | 7.00 | 3.89 | 1.14 | .51^***^ |  |  | 3.50 | 1.00 | | 4.28 | 1.16 | |
|  | AMI BM | 97 | 1.00 | 5.60 | 3.42 | 1.13 | .77^***^ | .73^***^ |  | 3.24 | 1.04 | | 3.60 | 1.19 | |
|  | AMI HM | 97 | 1.90 | 5.70 | 4.06 | 0.85 | .51^***^ | .40^***^ | .39^***^ | 4.26 | 0.86 | | 3.85 | 0.80 | |
|  |  |  |  |  |  |  |  |  |  |  |  | |  |  | |
| Study 2a | ASI BS | 85 | 1.09 | 6.45 | 3.78 | 1.10 |  |  |  | 3.66 | 1.13 | | 3.90 | 1.08 | |
|  | ASI HS | 85 | 1.09 | 6.55 | 3.80 | 1.19 | .50^***^ |  |  | 3.40 | 1.10 | | 4.20 | 1.15 | |
|  | AMI BM | 85 | 1.30 | 5.60 | 3.28 | 1.10 | .75^***^ | .72^***^ |  | 2.97 | 1.08 | | 3.59 | 1.06 | |
|  | AMI HM | 85 | 2.10 | 6.20 | 4.30 | 0.91 | .39^***^ | .25^*^ | .24^*^ | 4.48 | 1.02 | | 4.13 | 0.75 | |
|  |  |  |  |  |  |  |  |  |  |  |  | |  |  | |
| Study 2b | ASI BS | 92 | 1.27 | 5.82 | 3.89 | 0.99 |  |  |  | 3.81 | 1.00 | | 3.96 | 0.98 | |
|  | ASI HS | 92 | 1.18 | 6.36 | 3.95 | 1.09 | .58^***^ |  |  | 3.68 | 1.19 | | 4.25 | 0.89 | |
|  | AMI BM | 92 | 1.00 | 5.60 | 3.38 | 1.10 | .79^***^ | .72^***^ |  | 3.17 | 1.08 | | 3.61 | 1.08 | |
|  | AMI HM | 92 | 2.00 | 6.00 | 4.35 | 0.79 | .44^***^ | .34^**^ | .31^**^ | 4.53 | 0.77 | | 4.15 | 0.78 | |

*Note*. ^***^ *p* < .001; ^**^ *p* < .01; ^*^ *p* < .05

The present data, and in particular the divergences between scores of male and female respondents, are in line with the literature, which indicate generally higher scores in hostile sexism in male respondents. However, benevolent sexism scores highly diverge across countries: In an Italian study, Manganelli-Rattazzi, Volpato and Canova (2009) found no significant differences between male and female respondents for benevolent sexism, but male respondents scored higher in AMI-BM. These results are consistent with those found in the present study.

Scores of benevolent sexism (ASI-BS) did not significantly differ between male and female participants, *t*s < 1.02, *p*s > .31 for the three studies. Scores of benevolent attitude toward men (ASI-MS) did not significantly differ between male and female participants in Study 1, *t*(83) = 1.02, *p* = .31, but they were significantly higher in male participants in Study 2a, *t*(83) = 2.67, *p* = .009, and marginally significant in Study 2b, *t*(90) = 1.95, *p* = .054.

Male participants had on average significantly higher scores in hostile sexism (ASI-HS), *t*s > 2.62 and *p*s < .02 for the three studies. Female participants on the other hand had significantly higher scores in hostile attitude toward men (AMI-HM), *t*s > 2.35 and *p*s < .05 for the three studies.
