# Supplementary material for: Measuring objectification through the Body Inversion Paradigm: Methodological issues
Source: PLoS One. 2020 Feb 19;15(2):e0229161. doi: 10.1371/journal.pone.0229161 (PMC7031944; doi:10.1371/journal.pone.0229161)
Supplement: S3 File — (DOCX) [file pone.0229161.s003.docx]

**S3. Detailed Results**

Table 1C. *Test of the hypotheses for the three studies.*

|  | Study 1 | | Study 2a | | Study 2b | |
| --- | --- | --- | --- | --- | --- | --- |
|  | **accuracy** | latencies | **accuracy** | latencies | **accuracy** | latencies |
| Subj. Log-likelihood ratio | **Model 1 (intercept only)**  **LogLik = -3610.21**  **Model 2 (with subjects) LogLik = -3467.73**  **Chi-square(1) = 142.48,**  ***p* < .001** | Target model = -2865.038  Random model= -2479.855  Lratio = 770.3656, *p* <.001 | **Model 1 (intercept only)**  **LogLik = -1868.61**  **Model 2 (with subjects) LogLik = -1842.80**  **Chi-square(1) = 25.81**  ***p <.001*** | Target model = -2956.247  Random model=  -2071.517  Lratio = 1769.462, *p* <.001 | **Model 1 (intercept only)**  **LogLik = -1780.6**  **Model 2**  **LogLik = -1741.4,**  **Chi-square(1) = 39.2**  ***p < .001*** | Target model = -2920.121  Random model= -2234.5150  Lratio = 1371.944, *p* <.001 |
| Inversion effect | ***b* = 0.50 (0.06), *p < .0001*** | *b* = -0.10 (0.01), *p* < .0001 | ***b* = 0.66 (0.09), *p < .0001*** | *b* = -0.14 (.01), *p* < .0001 | ***b* = 0.43 (0.09), *p* < *.001*** | *b* = -0.08 (0.01), *p* < .0001 |
| **H1: stronger inversion for male targets** | | | | | | |
| Interaction:  Inversion * target sex | ***b* = -0.26 (0.13), *p* = .045** | *b* = 0.06 (0.02), *p* < .0001 | ***b* = 0.21 (0.18), *p* = *.22*** | b = 0.01 (.02),  *p* = .60 | ***b* = 0.32 (0.18), *p* = .08** | *b* = -0.03 (0.02), *p* = .15 |
| *Main effect of inversion:*  male targets | ***b* = 0.60 (0.08), *p* < .0001** | *b* = -0.13 (0.01), *p < .0001* | ***b* = 0.55 (0.12), *p* < *.0001*** | *b* = -0.14 (.02),  *p* < .0001 | ***b* = 0.27 (0.13), *p* = .04** | *b* = -0.07 (0.02), *p* < .001 |
| *Main effect of inversion:* female targets | ***b* = 0.35 (0.10), *p* = .00042** | *b* = -0.07 (0.01), *p <.0001* | ***b* = 0.77 (0.13), *p* = *.000*** | *b* = -0.13 (.02),  *p* < .0001 | ***b* = 0.58 (0.12), *p* < .001** | b = -0.10 (0.02), *p* < .001 |
| **H1b: difference in inversion effect for male and female targets is not influenced by gender of respondents** | | | | | | |
| Interaction:  Inversion * target sex* participant gender | ***b* = 0.35 (0.26), *p* = .18** | *b* = -0.06 (0.03), *p* = .07 | ***b* = -0.26 (0.35), *p* = .46** | *b* = -0.01 (0.05), *p* = .98 | ***b* = 0.09 (0.36), *p* = .80** | *b* = -0.10 (0.05), *p* = .03 |
| *Inversion effect: MPMT* | ***b* = 0.54 (0.12), *p* < .0001** | *b* = -0.13 (0.02), *p* < .0001 | ***b* = 0.57 (0.18), *p* < .0001** | *b* = -0.14 (0.02), *p* < .001 | ***b* = 0.25 (0.19), *p* = .19** | *b* = -0.04 (0.02), *p* = .10 |
| *Inversion effect: MPFT* | ***b* = 0.48 (0.15), *p* < .0001** | *b* = -0.11 (0.02), *p* < .0001 | ***b* = 0.64 (0.18), *p* < .0001** | *b* = -0.13 (0.03), *p* < .001 | ***b* = 0.62 (0.18), *p* < .0001** | *b* = -0.12 (0.03), *p* < .001 |
| *Inversion effect: FPMT* | ***b* = 0.64 (0.12), *p* < .0001** | *b* = -0.13 (0.02), *p* < .0001 | ***b* = 0.54 (0.16), *p* < .0001** | *b* = -0.15 (0.02), *p* < .001 | ***b* = 0.27 (0.17), *p* = .12** | *b* = -0.09 (0.02), *p* < .001 |
| *Inversion effect: FPFT* | ***b* = 0.23 (0.14), *p* =.08** | *b* = -0.04 (0.02), *p* = 0.004 | ***b* = 0.87 (0.17), *p* < .0001** | *b* = -0.14 (0.02), *p* < .001 | ***b* = 0.54 (0.17), *p* = .001** | *b* = -0.08 (0.02), *p* < .001 |
| Interaction:  Inversion * sex target, controlling for asymmetry | ***b* = -0.26 (0.13), *p* =.047** | *b* = 0.05 (0.02), *p* <.0001 | ***b* =0.21 (0.18), *p* = .22** | *b* = 0.01 (0.02), *p*  = .60 | ***b* = 0.32 (0.18), *p* = .08** | *b* = -0.03 (0.02), *p* = .15 |
| **Tests of moderation by characteristics of stimuli** | | | | | | |
| **H2 – asymmetry** | | | | | | |
| Main effect of asymmetry | ***b* = 0.44 (0.08), *p* < .0001** | *b* = -0.07 (0.01)*, p* < .0001 | ***b* =0.16 (0.10), *p* < .11** | *b* = -0.04 (0.01), *p* < .001 | ***b* = 0.21 (0.12), *p*= .08** | *b* = -0.03 (0.01), *p* < .001 |
| Interaction: Inversion * asymmetry | ***b* = -0.07 (.07) *p =* .32** | *b* = 0.02 (0.01), *p* = .012 | ***b* =0.23 (0.10), *p* = .023** | *b* = -0.02 (0.01), *p* = .14 | ***b* = 0.08 (0.10),  *p*= .41** | *b* = -0.03 (0.01), *p* = .03 |
| Interaction: Inversion* asymmetry * target sex | ***b* = -0.06 (0.18), *p* = .76** | *b* = 0.02 (0.02), *p* = .48 | ***b* =0.49 (0.21), *p* = .022** | *b* = -0.06 (0.01), *p* = .01 | ***b* = -0.10 (0.22), *p* = .65** | *b* = 0.01 (0.03), *p* = .80 |
| Interaction: Inversion* asymmetry * target sex * participant gender | ***b* = -0.60 (0.37), *p* = .11** | *b* = 0.01 (0.05), *p* = .82 | ***b* =0.30 (0.43), *p* = .48** | *b* = -0.09 (0.05), *p* = .07 | ***b* = 0.35 (0.45), *p* = .43** | *b* = 0.01 (0.05), *p* = .86 |
| *Inversion * asymmetry MPMT* | ***b* = 0.10 (0.14), *p* = .47** | *b* =- 0.01 (0.02), *p* = .88 | ***b* =0.01 (0.20), *p* = .97** | *b* = 0.04 (0.02), *p* = .04 | ***b* = 0.04 (0.21), *p* = .86** | *b* = -0.03 (0.02), *p* = .24 |
| *Inversion * asymmetry MPFT* | ***b* = -0.25 (0.16), *p* = .12** | *b* = 0.02 (0.02), *p* = .22 | ***b* =0.66 (0.26), *p* = .011** | *b* = -0.06 (0.03), *p* = .02 | ***b* = 0.15 (0.20), *p* = .47** | *b* = -0.03 (0.03), *p* = .28 |
| *Inversion * asymmetry FPMT* | ***b* = -0.02 (0.12), *p* = .81** | *b* = -0.00 (0.02), *p* = .96 | ***b* =0.05 (0.18), *p* = .77** | *b* = -0.01 (0.02), *p* = .70 | ***b* = 0.15 (0.20), *p* = .43** | *b* = -0.02 (0.02), *p* = .43 |
| *Inversion * asymmetry FPFT* | ***b* =-0.20 (0.15), *p* = .18** | *b* = 0.01 (0.02), *p* = 0.48 | ***b* =0.42 (0.21), *p* = .04** | *b* = -0.03 (0.02), *p* = .20 | ***b* = -0.05 (0.19), *p* = .80** | *b* = -0.02 (0.02), *p* = .32 |
| **H3: lower size of the inversion effect for more highly sexualized photographs** | | | | | | |
| Interaction:  Inversion * sexualization. | ***b* = -0.04 (.06), *p = .57*** | *b* = 0.02 (.01), *p* = .06 | ***b* =0.15 (0.10), *p* = .13** | *b* = -0.02 (0.01), *p* = .04 | ***b* = -0.02 (0.10), *p* = .84** | *b* = 0.02 (0.01), *p* = .07 |
| Inversion * sexualization * target sex | ***b* = -0.11 (0.13), *p = .43*** | *b* = -0.02 (0.02), *p* = .19 | ***b* =0.25 (0.30), *p* = .41** | *b* = -0.05 (0.04), *p* = .18 | ***b* = 0.07 (0.25), *p* = .77** | *b* = 0.07 (0.03), *p* = .03 |
| Inversion * sexualization * target sex * participant gender | ***b* = -0.49 (0.27), *p = .07*** | *b* = -0.01 (0.04), *p* = .74 | ***b* =0.60 (0.61), *p* = .32** | *b* = -0.06 (0.07), *p* = .43 | ***b* = -0.04 (0.50), *p* = .93** | *b* = -0.11 (0.06), *p* = .06 |
| Inversion * Sexualization MPMT | ***b* = -0.12 (0.12), *p = .28*** | *b* = 0.03 (0.02), *p* = .10 | ***b* =-0.09 (0.19), *p* = .63** | *b* = 0.02 (0.02), *p* = .47 | ***b* = -0.07 (0.20), *p* = .71** | *b* = 0.03 (0.02), *p* = .22 |
| Inversion * Sexualization MPFT | ***b* = -0.02 (0.14), *p = .88*** | *b* = 0.01 (0.02), *p* = .56 | ***b* =0.48 (0.25), *p* = .06** | *b* = -0.06 (0.03), *p* = .02 | ***b* = -0.07 (0.20), *p* = .74** | *b* = 0.05 (0.03), *p* = .04 |
| Inversion * Sexualization FPMT | ***b* = 0.16 (0.16), *p = .13*** | *b* = 0.02 (0.02), *p* = .33 | ***b* =0.10 (0.17), *p* = .56** | *b* = 0.00 (0.02), *p* = .88 | ***b* = -0.12 (0.18), *p* = .49** | *b* = -0.03 (0.02), *p* = .08 |
| Inversion * Sexualization FPFT | ***b* = -0.16 (0.14), *p = .26*** | *b* = 0.01 (0.02), *p* = .84 | ***b* =0.25 (0.19), *p* = .19** | *b* = -0.02 (0.02), *p* = .44 | ***b* =-0.11 (0.18), *p* = .53** | *b* = 0.06 (0.02), *p* = .003 |
| **H4: Effect of attractiveness** | | | | | | |
| Interaction:  Inversion * attractiveness | ***b* =-0.07 (.06), *p = .28*** | *b* = 0.02 (.01), *p* = .014 | ***b* =0.14 (0.08), *p* = .10** | *b* = 0.02 (0.01), *p* = .18 | ***b* = -0.08 (0.09), *p* = .39** | *b* = 0.01 (0.01), *p* = .41 |
| Interaction: Inversion * attractiveness * target sex | ***b* = 0.35 (.14), *p =* .011** | *b* = -0.02 (.02), *p* = .20 | ***b* =0.08 (0.18), *p* = .65** | *b* = 0.01 (0.02), *p* = .91 | ***b* = 0.14 (0.19), *p* = .46** | *b* = 0.03 (0.02), *p* = .16 |
| Interaction: Inversion * attractiveness * target sex * participant gender | ***b* = 0.17 (.28), *p = .55*** | *b* = 0.02 (.04), *p* = .54 | ***b* =0.09 (0.37), *p* = .80** | *b* = -0.02 (0.05), *p* = .73 | ***b* = -0.03 (0.38), *p* = .94** | *b* = -0.04 (0.05), *p* = .42 |
| Inversion * Attractiveness MPMT | ***b* = -0.24 (0.12), *p = .055*** | *b* =0.04 (0.02), *p* = .024 | ***b* =0.16(0.18), *p* = .36** | *b* = 0.01 (0.02), *p* = .52 | ***b* = -0.14 (0.20), *p* = .48** | *b* = 0.01 (0.02), *p* = .60 |
| Inversion * Attractiveness MPFT | ***b* = 0.16 (0.14), *p = .25*** | *b* = 0.02 (0.02), *p* = .35 | ***b* =0.32 (0.19), *p* = .09** | *b* = 0.01 (0.03), *p* = .83 | ***b* = -0.01 (0.18), *p* = .95** | *b* = 0.03 (0.03), *p* = .33 |
| Inversion * Attractiveness FPMT | ***b* = -0.13 (0.11), *p* = .28** | *b* = 0.01 (0.02), *p* = .66 | ***b* =0.01 (0.16), *p* = .96** | *b* = 0.02 (0.02), *p* = .47 | ***b* = -0.25 (0.18), *p* = .16** | *b* = -0.01 (0.02), *p* = .70 |
| Inversion * Attractiveness FPFT | ***b* = 0.11 (0.14), *p* = .43** | *b* = 0.01 (0.02), *p* = .62 | ***b* =0.07 (0.17), *p* = .67** | *b* = 0.02 (0.02), *p* = .31 | ***b* = -0.10 (0.17), *p* = .56** | *b* = 0.04 (0.02), *p* = .051 |
| **Tests of moderation by characteristics of perceivers** | | | | | | |
| **H5: (female targets): lower inversion associated with higher SCIAT scores** | | | | | | |
| Interaction:  Inversion * SCIAT | ***b* = -0.06 (0.07), *p* = .40** | *b* = -0.01 (0.01), *p* = .86 | ***b* =0.01 (0.09), *p* = .94** | *b* = 0.01 (0.01), *p* = .82 | ***b* = 0.09 (0.09), *p*= .32** | *b* = 0.01 (0.01), *p* = .30 |
| Inversion * SCIAT * target sex | ***b* = -0.06 (0.14), *p* = .66** | *b* = 0.01 (0.02), *p* = .37 | ***b* =0.21 (0.18), *p* = .23** | *b* = -0.02 (0.02), *p* = .41 | ***b* = 0.11 (0.18), *p* = .53** | *b* = 0.00 (0.02), *p* = .95 |
| Inversion * SCIAT * target sex * participant gender | ***b* = 0.34 (0.28), *p* = .22** | *b* = 0.02 (0.04), *p* = .55 | ***b* =0.16 (0.36), *p* = .66** | *b* = 0.08 (0.05), *p* = .09 | ***b* = -0.08 (0.37), *p* = .83** | *b* = 0.07 (0.05), *p* = .17 |
| Inversion * SCIAT MPMT | ***b* = -0.11 (0.12), *p* = .35** | *b* = -0.01 (0.02), *p* = .43 | ***b* =-0.26 (0.18), *p* = .15** | *b* = -0.02 (0.02), *p* = .34 | ***b* = -0.18 (0.19), *p* = .34** | *b* = -0.01 (0.02), *p* = .70 |
| Inversion * SCIAT MPFT | ***b* = 0.00 (0.15), *p* = .98** | *b* = 0.01 (0.02), *p* = .54 | ***b* =0.03 (0.19), *p* = .88** | *b* = 0.01 (0.03), *p* = .87 | ***b* = -0.12 (0.18), *p* = .51** | *b* = 0.02 (0.03), *p* = .38 |
| Inversion * SCIAT FPMT | ***b* = 0.05 (0.12), *p =* .70** | *b* = -0.01 (0.02), *p* = .78 | ***b* =0.03 (0.16), *p* = .86** | *b* = 0.04 (0.02), *p* = .07 | ***b* = 0.17 (0.17), *p* = .32** | *b* = -0.01 (0.02), *p* = .57 |
| Inversion * SCIAT  FPFT | ***b* = -0.16 (0.15), *p =* .24** | *b* = 0.01 (0.02), *p* = .87 | ***b* =0.19 (0.17), *p* = .28** | *b* = -0.02 (0.02), *p* = .48 | ***b* = 0.32 (0.17), *p* = .06** | *b* = -0.04 (0.02), *p* = .04 |
| **H6_BSH** | | | | | | |
| Interaction: Inversion * BSH | ***b* = 0.02 (0.06), *p =* .76** | *b* = 0.00 (.01), *p* = .59 | ***b* =-0.02 (0.08), *p* = .83** | *b* = 0.02 (0.01), *p* = .07 | ***b* = -0.02 (0.08), *p* = .81** | *b* = 0.00 (0.01), *p* = .71 |
| Interaction: Inversion * BSH* target sex | ***b* = 0.22 (0.12), *p* = .07** | *b* = 0.00 (.02), *p* = .95 | ***b* =-0.26 (0.17), *p* = .13** | *b* = 0.01 (0.02), *p* = .52 | ***b* = 0.21 (0.17) , *p* = .24** | *b* = 0.02 (0.02), *p* = .44 |
| Interaction: Inversion * BSH* target sex * participant gender | ***b* = -0.27(0.25), *p = .29*** | *b* = -0.04 (.03), *p* = .24 | ***b* =-0.29 (0.38), *p* = .45** | *b* = 0.00 (0.05), *p* = .99 | ***b* = -0.96 (0.38), *p* = .013** | *b* = 0.04 (0.05), *p* = .39 |
| *BSH*INVERSION MPMT* | ***b* = 0.14 (0.12), *p =* .21** | *b* = 0.04 (0.02), *p* = .048 | ***b* =-0.01 (0.18), *p* = .98** | *b* = 0.00 (0.02), *p* = .95 | ***b* = 0.44 (0.19), *p* = .02** | *b* = -0.04 (0.02), *p* = .13 |
| *BSH*INVERSION MPFT* | ***b* = 0.23 (0.12), *p =* .07** | *b* = 0.01 (0.02), *p* = .51 | ***b* =-0.44 (0.19), *p* = .023** | *b* = 0.01 (0.03), *p* = .63 | ***b* = 0.12 (0.18), *p* = .51** | *b* = -0.01 (0.03), *p* = .74 |
| *BSH*INVERSION FPMT* | ***b* = -0.39 (0.12), *p* = .011** | *b* = -0.02 (0.02), *p* = .16 | ***b* =0.18 (0.16), *p* = .25** | *b* = 0.03 (0.02), *p*  = .16 | ***b* = -0.53 (0.18), *p* = .003** | *b* = 0.01 (0.02), *p* = .45 |
| *BSH*INVERSION FPFT* | ***b* = 0.09 (0.14), *p* = .52** | *b* = -0.01 (0.02), *p* = .58 | ***b* =-0.25 (0.17), *p* = .76** | *b* = 0.05 (0.02), *p* = .046 | ***b* = 0.08 (0.17), *p* = .63** | *b* = 0.00 (0.02), *p* = .90 |
| **H6_BSV** | | | | | | |
| Interaction: Inversion * BSV | ***b* = 0.06 (0.07), *p* = .37** | *b* = 0.02 (.01), *p* = .02 | ***b* =-0.01 (0.09), *p* = .90** | *b* = 0.02 (0.01), *p* = .11 | ***b* = 0.16 (0.09), *p* = .08** | *b* = -0.01 (0.01), *p* = .42 |
| Interaction: Inversion * BSV* target sex | ***b* = 0.03 (0.13), *p* = .84** | *b* = 0.00 (.02), *p =* .95 | ***b* =-0.32 (0.18), *p* = .08** | *b* = 0.01 (0.02), *p* = .57 | ***b* = 0.18 (0.18), *p* = .32** | *b* = 0.03 (0.02), *p* = .16 |
| Interaction: Inversion * BSV* target sex * participant gender | ***b* = 0.09 (0.29), *p* = .75** | *b* = -.04 (.04), *p p= .19* | ***b* =-0.05 (0.37), *p* = .89** | *b* = 0.06 (0.05), *p* = .22 | ***b* = -0.95 (0.38), *p* = .012** | *b* = 0.03 (0.05), *p* = .50 |
| *BSV*INVERSION MPMT* | ***b* = 0.12 (0.12), *p* = .32** | *b* = 0.03 (0.02), *p* = .06 | ***b* =0.08 (0.18), *p* = .68** | *b* = 0.01 (0.02), *p* = .56 | ***b* = 0.42 (0.01), *p* < .001** | *b* = -0.05 (0.02), *p* = .027 |
| *BSV*INVERSION MPFT* | ***b* = 0.23 (0.15), *p* = .11** | *b* = 0.01 (0.02), *p* = .56 | ***b* =-0.30 (0.19), *p* = .11** | *b* = 0.05 (0.03), *p* = .04 | ***b* = 0.18 (0.18), *p* = .30** | *b* = -0.02 (0.03), *p* = .51 |
| *BSV*INVERSION FPMT* | ***b* = -0.07 (0.12), *p* = .54** | *b* = 0.01 (0.02), *p* = .92 | ***b* =0.19 (0.16), *p* = .23** | *b* = 0.02 (0.02), *p* = .46 | ***b* = -0.29 (0.18), *p* = .12** | *b* = 0.01 (0.02), *p* = .46 |
| *BSV*INVERSION FPFT* | ***b* = -0.08 (0.15), *p* = .61** | *b* = 0.02 (0.02), *p* = .16 | ***b* =-0.10 (0.18), *p* = .57** | *b* = 0.00 (0.02), *p* = .92 | ***b* = 0.35 (0.17), *p* = .04** | *b* = 0.02 (0.02), *p* = .47 |
| **H7_BS** | | | | | | |
| Interaction: Inversion * BS | ***b* = -0.03 (0.06), *p* = .63** | *b* = -0.01 (.01), *p* = .27 | ***b* =-0.05 (0.09), *p* = .54** | *b* = 0.01 (0.01), *p* = .25 | ***b* = 0.01 (0.09), *p* = .93** | *b* = -0.01 (0.01), *p* = .62 |
| Interaction: Inversion * BS * target sex | ***b* = 0.13 (0.13), *p* = .30** | *b* = -0.02 (.02), *p* = .19 | ***b* =-0.15 (0.17), *p* = .38** | *b* = 0.01 (0.02), *p* = .81 | ***b* = -0.06 (0.18), *p* = .73** | *b* = 0.04 (0.02), *p* = .07 |
| Interaction: Inversion * BS * target sex * participant gender | ***b* = 0.31 (0.26), *p* = .23** | *b* = -0.03 (.03), *p* = .36 | ***b* =-0.24 (0.35), *p* = .49** | *b* = -0.01 (0.05), *p* = .90 | ***b* = -0.52 (0.39), *p* = .18** | *b* = 0.00 (0.05), *p* = .93 |
| *BS*INVERSION MPMT* | ***b* = 0.06 (0.12), *p* = .65** | *b* = -0.01 (0.02), *p* = .67 | ***b* =0.04 (0.18), *p* = .84** | *b* = -0.01 (0.02), *p* = .81 | ***b* = 0.37 (0.23), *p* = .10** | *b* = -0.03 (0.02), *p* = .20 |
| *BS*INVERSION MPFT* | ***b* = 0.32 (0.14), *p* = .018** | *b* = -0.05 (0.02), *p* = .006 | ***b* =-0.24 (0.19), *p* = .31** | *b* = 0.00 (0.03), *p* = .93 | ***b* = -0.02 (0.20), *p* = .93** | *b* = 0.01 (0.03), *p* = .61 |
| *BS*INVERSION FPMT* | ***b* = -0.23 (0.12), *p* = .047** | *b* = 0.01 (0.02), *p* = .47 | ***b* =-0.01 (0.15), *p* = .99** | *b* = 0.02 (0.02), *p* = .27 | ***b* = -0.15 (0.17), *p* = .38** | *b* = -0.03 (0.02), *p* = .15 |
| *BS*INVERSION FPFT* | ***b* = -0.28 (0.14), *p* = .044** | *b* = 0.01 (0.02), *p* = .66 | ***b* =-0.03 (0.17), *p* = .84** | *b* = 0.03 (0.02), *p* = .17 | ***b* = -0.04 (0.17), *p* = .83** | *b* = 0.02 (0.02), *p* = .33 |
| **H7_HS** | | | | | | |
| Interaction: Inversion * HS | ***b* = -0.03 (0.06), *p = .60*** | *b* = -0.00 (.02), *p* = .92 | ***b* =-0.01 (0.09), *p* = .94** | *b* = 0.01 (0.01), *p* = .54 | ***b* = -0.07 (0.09), *p* = .45** | *b* = 0.01 (0.01), *p* = .26 |
| Interaction: Inversion * HS * target sex | ***b* = 0.04 (0.12), *p = .77*** | *b* = -0.03 (.02), *p* = .10 | ***b* =-0.04 (0.17), *p* = .81** | *b* = 0.01 (0.02), *p* = .61 | ***b* = 0.23 (0.18), *p* = .20** | *b* = 0.01 (0.02), *p* = .55 |
| Interaction: Inversion * HS * target sex * participant gender | ***b* = 0.53 (0.26), *p* = .041** | *b* = -0.02 (.04), *p* = .52 | ***b* =-0.12 (0.37), *p* = .74** | *b* = -0.01 (0.05), *p* = .92 | ***b* = -0.96 (0.44), *p* = .028** | *b* = -0.01 (0.05), *p* = .85 |
| *HS*INVERSION MPMT* | ***b* = 0.07 (0.11), *p = .56*** | *b* = 0.01 (0.02), *p* = .52 | ***b* =0.10 (0.18), *p* = .57** | *b* = -0.02 (0.02), *p* = .29 | ***b* = 0.38 (0.22), *p* = .08** | *b* = 0.00 (0.02), *p* = .83 |
| *HS*INVERSION MPFT* | ***b* = 0.25 (0.12), *p* = .04** | *b* = -0.02 (0.02), *p* = .25 | ***b* =0.04 (0.19), *p* = .84** | *b* = -0.01 (0.02), *p* = .67 | ***b* = 0.01 (0.19), *p* = .94** | *b* = 0.01 (0.03), *p* = .60 |
| *HS*INVERSION FPMT* | ***b* = -0.15 (0.11), *p = .17*** | *b* = 0.02 (0.02), *p* = .32 | ***b* =-0.07 (0.16), *p* = .63** | *b* = 0.02 (0.02), *p* = .35 | ***b* = -0.48 (0.18), *p* = .007** | *b* = 0.00 (0.02), *p* = .96 |
| *HS*INVERSION FPFT* | ***b* = -0.47 (0.14), *p =* .0005** | *b* = 0.01 (0.02), *p* = .51 | ***b* =-0.02 (0.17), *p* = .89** | *b* = 0.03 (0.02), *p* = .14 | ***b* = 0.04 (0.17), *p* = .80** | *b* = 0.04 (0.02), *p* = .07 |
| **H7_BM** | | | | | | |
| Interaction: Inversion * BM | ***b* = 0.03 (0.06), *p = .59*** | *b* = 0.00 (.01), *p* = .99 | ***b* =0.03 (0.09), *p* = .72** | *b* = 0.01 (0.01), *p* = .63 | ***b* = 0.09 (0.09), *p* = .34** | *b* = -0.01 (0.01), *p* = .40 |
| Inversion * BM * target sex | ***b* = 0.05 (0.13), *p = .68*** | *b* = -0.02 (.02), *p* =.16 | ***b* =-0.06 (0.17), *p* = .73** | *b* = 0.01 (0.02), *p* = .54 | ***b* = -0.01 (0.18), *p* = .96** | *b* = 0.04 (0.02), *p* = .11 |
| Inversion * BM * sex of target * part gender | ***b* = 0.54 (0.27), *p* = .04** | *b* = -0.01 (.03), *p* =.75 | ***b* =-0.57 (0.37), *p* = .12** | *b* = 0.03 (0.05), *p* = .57 | ***b* = -0.29 (0.39), *p* = .46** | *b* = 0.01 (0.05), *p* = .80 |
| *BM*INVERSION MPMT* | ***b* = 0.13 (0.12), *p = .29*** | *b* = 0.01 (0.02), *p* = .57 | ***b* =0.17 (0.18), *p* = .33** | *b* = -0.02 (0.02), *p* = .32 | ***b* = 0.44 (0.00), *p* < .001** | *b* = -0.05 (0.02), *p* = .02 |
| *BM*INVERSION MPFT* | ***b* = 0.39 (0.14), *p =* .004** | *b* = -0.02 (0.02), *p* = .34 | ***b* =-0.14 (0.19), *p* = .45** | *b* = 0.01 (0.03), *p* = .75 | ***b* = 0.20 (0.19), *p* = .29** | *b* = 0.00 (0.03), *p* = .98 |
| *BM*INVERSION FPMT* | ***b* = -0.11 (0.12), *p = .34*** | *b* = 0.01 (0.02), *p* = .40 | ***b* =-0.04 (0.16), *p* = .80** | *b* = 0.01 (0.02), *p*  = .55 | ***b* = -0.09 (0.17), *p* = .58** | *b* = -0.02 (0.02), *p* = .36 |
| *BM*INVERSION FPFT* | ***b* = -0.39 (0.14), *p* = .007** | *b* = 0.00 (0.02), *p* = .92 | ***b* =0.19 (0.18), *p* = .28** | *b* = 0.01 (0.02), *p* = .53 | ***b* = -0.01 (0.17), *p* = .94** | *b* = 0.03 (0.02), *p* = .22 |
| **H7_HM** | | | | | | |
| Interaction: Inversion * HM | ***b* = -0.03 (0.06), *p* = .64** | *b* = 0.00 (.01), *p* = .85 | ***b* =-0.01 (0.09), *p* = .93** | *b* = 0.01 (0.01), *p* = .51 | ***b* = -0.04 (0.09), *p* = .62** | *b* = 0.01 (0.01), *p* = .45 |
| Interaction: Inversion * HM * target sex | ***b* = 0.10 (0.13), *p* = .44** | *b* = -0.01 (.02), *p* = .68 | ***b* =-0.12 (0.17), *p* = .47** | *b* = 0.05 (0.02), *p* = .046 | ***b* = -0.01 (0.18), *p* = .98** | *b* = 0.05 (0.02), *p* = .04 |
| Interaction: Inversion * HM * target sex * participant gender | ***b* = 0.30 (0.27), *p* = .27** | *b* = -0.02 (.03), *p* = .63 | ***b* =-0.12 (0.37), *p* = .74** | *b* = -0.06 (0.05), *p* = .22 | ***b* = -0.89 (0.38), *p* = .02** | *b* = 0.01 (0.05), *p* = .84 |
| *HM*INVERSION MPMT* | ***b* = 0.04 (0.12), *p* = .72** | *b* = 0.00 (0.02), *p* = .96 | ***b* =0.04 (0.17), *p* = .80** | *b* = -0.01 (0.02), *p* = .72 | ***b* = 0.25 (0.20), *p* = .21** | *b* = -0.02 (0.02), *p* = .43 |
| *HM*INVERSION MPFT* | ***b* = 0.35 (0.14), *p* = 015** | *b* = -0.02 (0.02), *p* = .16 | ***b* =-0.15 (0.19), *p* = .41** | *b* = 0.00 (0.03), *p* = .97 | ***b* = -0.22 (0.18), *p* = .24** | *b* = 0.02 (0.03), *p* = .39 |
| *HM*INVERSION FPMT* | ***b* = -0.21 (0.12), *p* = .07** | *b* = 0.01 (0.02), *p* = .54 | ***b* =0.06 (0.16), *p* = .71** | *b* = -0.02 (0.02), *p* = .36 | ***b* = -0.28 (0.18), *p* = .12** | *b* = 0.00 (0.02), *p* = .96 |
| *HM*INVERSION FPFT* | ***b* = -0.19 (0.14), *p* = .17** | *b* = 0.00 (0.02), *p* = .88 | ***b* =-0.06 (0.17), *p* = .73** | *b* = 0.06 (0.02), *p* = .014 | ***b* = 0.10 (0.17), *p* = .56** | *b* = 0.03 (0.02), *p* = .13 |
| **Q1 – ASYMMETRY, BUILDINGS** | | | | | | |
| Inversion | ***b* = 0.15 (.09), *p =* .09** | *b* = -0.02 (.01) , *p* = .12 | ***b* =0.01 (0.09), *p* = .93** | *b* = -0.01 (0.01), *p* = .51 | ***b* =0.22 (0.09), *p* = .016** | *b* = -0.01 (0.01), *p* = .51 |
| Main effect of asymmetry on recognition | ***b* = 0.25 (.11), *p* = .03** | *b* = -0.04 (.01), *p* < .001 | ***b* =0.31 (0.10), *p* = .001** | *b* = -0.05 (0.01), *p* < .000 | ***b* =0.19 (0.12), *p* = .11** | *b* = -0.05 (0.01), *p* < .001 |
| Asymmetry * Inversion | ***b* = -0.08 (.09), *p* = .39** | *b* = -0.01 (.01), *p* =*.54* | ***b* =-0.17 (0.10), *p* = .09** | *b* = -0.01 (0.01), *p* = .30 | ***b* =0.02 (0.09), *p* = .80** | *b* = -0.01 (0.01), *p* = .30 |

*Note*. We considered only Italian participants.

MPMT = male participants, male targets; MPFT = male participants, female targets; FPMT = female participants, male targets; FPFT = female participants, female targets; SCIAT = Single Category Implicit Association Test; BSH = body shame; BSV = body surveillance.

Categorical predictors were dummy coded as follows:

Inversion: 0 = Inverted, 1 = Upright;

Target sex: 0 = Male, 1 = Female;

Participant gender: 0 = Female, 1 = Male.

Note for the interpretation of the impact of moderating variables on the inversion effect as measured by latencies:

We expected a negative impact of target orientation on latencies, because upright photos should be recognized faster, hence implying lower reaction times. Therefore, a negative sign of the interaction between a variable and target orientation for latencies indicates that this variable increases the inversion effect, while a positive sign of the interaction indicates that the variable decreases the inversion effect.

**Four-way Interactions**

As research suggests that male and female individuals objectify women for different reasons (Vaes, Paladino, & Puvia, 2011), we deemed it important to check whether the effects of the moderators attractiveness and sexualization of the targets, automatic woman-human association, self-sexualization and gender attitudes of the participants affected the inversion effect differently, depending on the gender of the participants. Therefore, for each moderator we tested the 4-way interaction with target orientation, target sex and participant gender. In the cases in which the 4-way interaction was significant, we further tested the impact of the moderator on the inversion through a series of four moderator * target orientation interactions: one for each of the conditions created by target sex * participant gender.

The results of these analyses are displayed on Table 1C above and briefly discussed here

Sexualization (H3). No significant 4-way interaction emerged.

Attractiveness (H4). No significant 4-way interaction emerged.

Automatic woman-human association (H5). No significant 4-way interaction emerged.

Self-objectification (H6). We separately investigated the moderating effect of two different dimensions of self-objectification: BSH and BSV.

For BSH, a significant 4-way interaction emerged only in Study 3. For male participants, the higher the BSH score, the lower the alleged objectification of male targets, while one would expect that participants characterized by higher body shame would objectify participants of the same sex to a greater extent. For female participants, higher BSH scores were associated with lower inversion scores and, hence, higher alleged objectification of male targets. In this case, the relationship between higher self-objectification and higher objectification of targets is consistent with expectations, but unexpectedly, it emerged only for targets of the opposite sex. Also for BSV, a significant 4-way interaction emerged only in Study 3. Participants with higher BSV scores, in Study 3, showed higher inversion when reacting to targets of their own sex. This would suggest that, contrary to expectations, participants with higher levels of self-objectification objectify own-sex targets less than other participants.

Gender attitudes (H7). We separately investigated the moderating effect of four different dimensions: BS, HS, BM, and HM.

No 4-way interaction was significant, in either study, for BS.

For HS, the 4-way interaction was significant in Study 1 and in Study 2b, but the direction of the interaction effect was opposite. It was not significant in Study 2a. A closer inspection revealed a highly inconsistent pattern of results: In Study 1 (original sexualized photos used in Bernard et al., 2012) HS was significant only when female targets were involved. Specifically, male participants with higher HS scores showed stronger inversion effects for female targets, while female participants with higher HS scores showed weaker inversion effects for female targets.

These results would suggest that men characterized by higher levels of HS objectify sexualized women less and that women characterized by higher HS objectify sexualized women more, as compared to individuals characterized by lower levels of HS and BM. The influence of sexism on the size of the inversion effect for women was not unexpected since women are the typical target of objectification. Also, the result that more-sexist women objectified other women more was in line with our predictions. However, one would expect that men with higher sexist beliefs would objectify sexualized women more, not less as found, than other men.

In Study 2b (non-sexualized photos) on the other hand, HS was a significant moderator only for female participants who reacted to male targets: More specifically, female participants with higher HS scores had lower inversion effects when they reacted to male targets. If we take the IBRT as a measure of objectification, this result suggests that the higher the woman’s hostile sexism, the more they objectify non-sexualized men.

For BM, a significant 4-way interaction emerged only in Study 1, where (similarly to HS) BM moderated the inversion effect only for female targets: For male participants reacting to female targets, higher BM levels were associated with stronger inversion effects, while for female participants reacting to female targets, higher BM levels were associated with lower inversion effects.

Finally, the 4-way interaction involving HM was significant only in Study 2b; however, when we tested whether HM moderated the inversion effect in the four cells created by target sex and participant gender, we found no significant effect. Therefore, the four-way interaction is most probably attributable to random noise.

**Overall**: Also the analysis of 4-way interactions failed to provide clear evidence in favor of the validity of the IBRT. Significant interactions emerged only for the moderators concerning self-objectification and gender attitudes. However the patterns of results were inconsistent across studies and theoretically unexpected.

In evaluating these results, it is important to notice that, to maximize our ability to detect any evidence of construct validity, we conducted various tests for each hypothesis. The side-effect of such a fine mesh net is the increased risk of Type-I error, and it is therefore plausible that these inconsistent effects are the result of random noise (see McShane, Gal, Gelman, Robert, & Tackett, 2019 for a recent suggestion to weight the *p*-level against the plausibility of the results).

**References**

McShane, B, Gal, D, Gelman, A, Robert, C, Tackett, JL. Abandon statistical significance. Am Stat. 2019; Available from: doi: 10.1080/00031305.2018.1527253

Vaes, J., Paladino, P., & Puvia, E. (2011). Are sexualized women complete human beings? Why men and women dehumanize sexually objectified women. *European Journal of Social Psychology*, *41*(6), 774-785.
