# Supplementary material for: Measuring objectification through the Body Inversion Paradigm: Methodological issues
Source: PLoS One. 2020 Feb 19;15(2):e0229161. doi: 10.1371/journal.pone.0229161 (PMC7031944; doi:10.1371/journal.pone.0229161)
Supplement: S4 File — (DOCX) [file pone.0229161.s004.docx]

**S4. Sensitivity analysis.**

We conducted a sensitivity analysis for studies 1 and 2a (we did not perform the sensitivity analysis for study 2b because it had the same identical structure as study 2a, the only difference being the specific pictures presented to participants), in order to establish the value of the minimum effect size that the present paper was able to capture. Indeed, a critical reader might wonder whether the moderation effects observed here were volatile because of an insufficient number of observations.

Using the simr package (Green & MacLeod, 2016) in R, we ran a Monte Carlo simulation, which is considered the gold standard for power analysis (Abraham & Russell, 2008; Arnold, Hogan, Colford, & Hubbard, 2011). Because Monte Carlo analyses involve a large number of randomly simulated data samples from specified populations, researchers can empirically evaluate sampling distributions for effects of interest.

Statistical power was estimated based on 400 simulations. Its target statistical value was 80%, and the R package 'SIMR' (Green and MacLeod, 2016) was used. The power analysis was performed for a generalized linear mixed model (GLMM), namely for a binomial logistic regression with completely crossed-random effects, *i.e.* every person looks at each target and each target is submitted to every person. Crossed random-effects models extend multilevel models, where subjects are nested into items. Estimation procedures are analogous (Bryk & Raudenbush, 1992; Hox, Moerbeek & van de Shoot, 2017). The analysis we conducted is an extension of multilevel modeling based on binomial logistic regression.

**Effect size computation** was based on the *odds ratio* (OR), an unstandardized effect size statistic, which tells the direction and the strength of the relationship between the predictor and the odds that the criterion is equal 1, i.e. that the answer to the test is correct. The OR is the ratio between the odds of the event when the predictor assumes value 1, and the odds of the event when the predictor assumes value 0. In other words, the effect of changing the predictor from 0 to 1 changes the odds of getting 1 in the outcome, increasing them (if OR >1) or decreasing them (if OR<1).

The OR for an independent variable in logistic regression represents the change in the odds for a unitary increase in that variable, holding all other variables constant.

Based on the OR, we computed Cohen’s d, which is a widely known index of effect size, with the following formula:

*d* = log(OR) × $\sqrt{3}$ /*π*

(see: Borenstein, M., Hedges, L. V., Higgins, J. P. T., and Rothstein, H. R. (2009). Introduction to Meta-Analysis. John Wiley and Sons, ISBN: 978-0-470-05724-7)*.*

In the tables hereafter, we present the observed (estimated) effect size, and the required effect size, in terms of beta, odds ratio, and Cohen’s d, for the two studies.

**Table 1D. Study 1**

|  | observed effect size (estimate) | | | sensitivity analysis: required effect size | | | Power (1-$\beta)$ 95%CI |
| --- | --- | --- | --- | --- | --- | --- | --- |
| tested effect | Beta  (regression cefficient) | Odds Ratio | Cohen’s d | Beta | Odds Ratio | Cohen’s d |  |
| Inversion Effect | 0.50 | 1.67 | 0.28 | 0.17 | 1.19 | 0.10 | .81 (.77, .84) |
| Inversion  * Target Sex | -0.26 | 0.77 | 0.14 | -0.38 | 0.68 | 0.21 | .82 (.78, .85) |
| Inversion  * Target Asymmetry | -0.07 | 0.92 | 0.04 | -0.22 | 0.80 | 0.12 | .82 (.78, .86) |
| Inversion * Target Asymmetry * Target Sex | -0.06 | 0.94 | 0.03 | -0.50 | 0.61 | 0.28 | .81 (.77, .85) |
| Inversion  * Target Sexualization | -0.04 | 0.96 | 0.02 | - 0.19 | 0.82 | 0.11 | .81 (.77, .85) |
| Inversion  * Target Sexualization * Target sex | -0.11 | 0.90 | 0.06 | - 0.40 | 0.67 | 0.22 | .83 (.78, .86) |
| Inversion  * Target Attractiveness | - 0.07 | 0.93 | 0.04 | - 0.18 | 0.84 | 0.10 | .81 (.77, .85) |
| Inversion  * Target Attractiveness * Target Sex | 0.35 | 1.42 | 0.19 | 0.38 | 1.46 | 0.21 | .83 (.79, .86) |
| Inversion  * SC-IAT | -0.06 | 0.9 | 0.03 | - 0.19 | 0.83 | 0.11 | .82 (.79, .86) |
| Inversion  * SC-IAT * Target Sex | -0.06 | 0.94 | 0.03 | - 0.36 | 0.70 | 0.20 | .81 (.77, .85) |
| Inversion  * BSH | 0.02 | 1.02 | 0.01 | 0.18 | 1.20 | 0.10 | .80 (.76, .84) |
| Inversion  * BSH * Target Sex | 0.22 | 1.25 | 0.12 | 0.39 | 1.48 | 0.22 | .82 (.78, .85) |
| Inversion  * BSV | 0.06 | 1.06 | 0.03 | 0.19 | 1.21 | 0.10 | .81 (.77, .85) |
| Inversion  * BSV * Target sex | 0.03 | 1.03 | 0.01 | 0.37 | 1.45 | 0.20 | .81 (.77, .84) |
| Inversion  * BS | -0.03 | 0.97 | 0.02 | - 0.18 | 0.84 | 0.10 | .80 (.76, .84) |
| Inversion  * BS * Target Sex | 0.13 | 1.14 | 0.07 | 0.37 | 1.45 | 0.20 | .80 (.76, 84) |
| Inversion  * HS | -0.03 | 0.97 | 0.02 | - 0.18 | 0.84 | 0.10 | .81 (.76, .84) |
| Inversion  * HS * Targer Sex | 0.04 | 1.04 | 0.02 | 0.38 | 1.46 | 0.21 | .80 (.76, .84) |
| Inversion  * BM | 0.03 | 1.03 | 0.02 | 0.19 | 1.21 | 0.10 | .83 (.74, .90) |
| Inversion  * BM * Target Sex | 0.05 | 1.05 | 0.03 | 0.38 | 1.46 | 0.21 | .81 (.71, .88) |
| Inversion  * HM | -0.03 | 0.97 | 0.02 | - 0.19 | 0.83 | 0.10 | .84 (.80, .87) |
| Inversion  * HM * Target Sex | 0.10 | 1.10 | 0.05 | 0.38 | 1.46 | 0.21 | .84 (.80, .88) |

**Table 2. Study 2a**

|  | observed effect size (estimate) | | | sensitivity analysis: required effect size | | | Power (1-$\beta)$95%CI |
| --- | --- | --- | --- | --- | --- | --- | --- |
| tested effect | Beta  (regression coefficient) | Odds Ratio | Cohen’s d | Beta | Odds Ratio | Cohen’s d |  |
| Inversion Effect | 0.66 | 1.93 | 0.36 | 0.23 | 1.26 | 0.13 | .81 (.77, .85) |
| Inversion  * Target Sex | 0.21 | 1.24 | 0.12 | 0.50 | 1.64 | 0.27 | .82 (.78, .85) |
| Inversion  * Target Asymmetry | 0.23 | 1.25 | 0.12 | 0.27 | 1.31 | 0.15 | .82 (.78, .85) |
| Inversion * Target Asymmetry * Target Sex | 0.49 | 1.61 | 0.26 | 0.59 | 1.80 | 0.33 | .80 (.76, .84) |
| Inversion  * Target Sexualization | 0.15 | 1.15 | 0.08 | 0.26 | 1.30 | 0.14 | .80 (.76, .84) |
| Inversion  * Target Sexualization * Target sex | 0.25 | 1.28 | 0.14 | 0.90 | 2.46 | 0.50 | .80 (.76, .84) |
| Inversion  * Target Attractiveness | 0.14 | 1.15 | 0.08 | 0.25 | 1.28 | 0.14 | .82 (.78, .86) |
| Inversion  * Target Attractiveness * Target Sex | 0.08 | 1.06 | 0.03 | 0.52 | 1.68 | 0.29 | .81 (.76, .84) |
| Inversion  * SC-IAT | 0.01 | 1.01 | <0.01 | 0.25 | 1.28 | 0.14 | .81 (.77, .85) |
| Inversion  * SC-IAT * Target Sex | 0.21 | 1.24 | 0.12 | 0.49 | 1.63 | 0.27 | .80 (.76, .84) |
| Inversion  * BSH | -0.02 | 0.98 | <0.01 | - 0.25 | 0.78 | 0.14 | .83 (.79, .87) |
| Inversion  * BSH * Target Sex | -0.26 | 0.77 | 0.14 | - 0.50 | 0.61 | 0.28 | .80 (.76, .84) |
| Inversion  * BSV | -0.01 | 0.99 | 0.01 | - 0.25 | 0.78 | 0.14 | .81 (.77, .85) |
| Inversion  * BSV * Target sex | -0.32 | 0.72 | 0.18 | - 0.51 | 0.60 | 0.28 | .80 (.76, .84) |
| (only female targets)  Inversion  * BSV | -0.19 | 0.99 | 0.01 | - 0.38 | 0.68 | 0.21 | .80 (.76, .84) |
| Inversion  * BS | -0.05 | 0.95 | 0.03 | - 0.26 | 0.77 | 0.14 | .82 (.78, .86) |
| Inversion  * BS * Target Sex | -0.15 | 0.86 | 0.08 | - 0.51 | 0.60 | 0.28 | .81 (.77, .85) |
| Inversion  * HS | -0.01 | 0.99 | <0.01 | - 0.25 | 0.78 | 0.14 | .82 (.78, .85) |
| Inversion  * HS * Targer Sex | -0.04 | 0.96 | 0.02 | - 0.49 | 0.61 | 0.27 | .80 (.76, .84) |
| Inversion  * BM | 0.03 | 1.03 | 0.02 | 0.26 | 1.30 | 0.14 | .83 (.79, .87) |
| Inversion  * BM * Target Sex | -0.06 | 0.94 | 0.03 | - 0.50 | 0.61 | 0.28 | .82 (.78, .86) |
| Inversion  * HM | -0.01 | 0.99 | <0.01 | - 0.25 | 0.78 | 0.14 | .82 (.78, .85) |
| Inversion  * HM * Target Sex | -0.12 | 0.88 | 0.07 | - 0.49 | 0.61 | 0.27 | .80 (.76, .84) |
