# Supplementary material for: Measuring objectification through the Body Inversion Paradigm: Methodological issues
Source: PLoS One. 2020 Feb 19;15(2):e0229161. doi: 10.1371/journal.pone.0229161 (PMC7031944; doi:10.1371/journal.pone.0229161)
Supplement: S5 File — (DOCX) [file pone.0229161.s005.docx]

**S5. Additional analyses on asymmetry in the bust area**

As an additional check on the role played by stimulus asymmetry, for each photo, we also computed an index of asymmetry of the bust area, as it can be considered an “area of interest” containing the secondary sexual characters (breasts, pelvis, hips). This new bust asymmetry index was the sum of the distance scores for navel, shoulders, and hips (we thank an anonymous reviewer for the suggestion to investigate the possibility of specific effects of asymmetry in this area of interest).

First, we checked the presence of differences between female and male stimuli. Table 1 reports means and standard deviations for the stimuli used in each of the studies, for male and female targets. When the attention is restricted to the bust area, the differences in asymmetry between male and female targets in Study 1 (i.e., the original materials used by Bernard and colleagues, 2012) are still present and significant, *t*(46)= 2.76, *p* = 0.008, *d* = 0.37.

In Study 2a and in Study 2b the differences between male and female targets are not significant, for both sets, *t*s < 1, *p*s > .45, Cohen’s *d*s < 0.10.

**Means and Standard Deviations of the Bust Asymmetry index for the sets of stimuli of Study 1, Study 2a, and Study 2b, depending on the sex of the target.**

| study | Sex | Mean | Standard Deviation | N |
| --- | --- | --- | --- | --- |
| 1 | male | 101.58 | 48,90 | 24 |
|  | female | 138.95 | 44,76 | 24 |
|  | Whole set | 120,26 | 50,07 | 48 |
| 2 a | male | 63,22 | 31,67 | 12 |
|  | female | 56,47 | 19,26 | 12 |
|  | Whole set | 59,85 | 25,87 | 24 |
| 2 b | male | 63,82 | 32,93 | 12 |
|  | female | 73,02 | 25,74 | 12 |
|  | Whole set | 68,42 | 29,28 | 24 |

Next, we run new analyses with the bust asymmetry index, identical to those we performed with the overall asymmetry index.

These analyses confirmed that higher asymmetry, also when restricted to the bust area, was associated with an **increase in accuracy of responses,** indicating a higher level of accuracy for more asymmetric stimuli. The effect was not significant for Study 2b, but the pattern was the same for the three studies.

**Bust asymmetry effect**

| **Study 1** | *b* = 0.18 | *SE* = 0.09 | *p* = .048 |
| --- | --- | --- | --- |
| **Study 2a** | *b* = 0.21 | *SE* = 0.09 | *p* = .02 |
| **Study 2b** | *b* = 0.11 | *SE* = 0.11 | *p* =.28 |

As concerns the interaction between bust asymmetry and target orientation, results were inconsistent across studies, indicating that in Study 1 higher bust asymmetry was associated with lower inversion effect (which would be consistent with the hypothesis), but in Study 2a the opposite result emerged significant, and in Study 2b the effect failed to reach statistical significance.

**Bust asymmetry * target orientation effect**

| **Study 1** | *b* = -0.19 | *SE* = 0.06 | *p* = .001 |
| --- | --- | --- | --- |
| **Study 2a** | *b* = 0.21 | *SE* = 0.09 | *p* = .03 |
| **Study 2b** | b = 0.11 | *SE* = 0.10 | *p* = .28 |

The higher order interactions target orientation * asymmetry-bust * target sex, and target orientation * asymmetry-bust * target sex * participant gender were not significant, *ps* > .50 in the three studies, with the exception of the target orientation * bust asymmetry * target sex interaction, which was significant in Study 2a, *b* = 0.56, *SE* = 0.21, *p* < 0.001, showing similar results as those we obtained with the index of overall asymmetry based on all asymmetry data points

Finally, the results for the target orientation * target sex interaction, when controlling for the bust asymmetry, were inconsistent between studies, and showed an overall pattern that was very similar to that reported in the manuscript, in which we used the overall index of asymmetry. Indeed, in Study 1 we found a negative interaction, indicating that even controlling for bust asymmetry female target showed lower inversion effects (note that the interaction was not significant, but the size of the regression coefficient was very similar to the one obtained using the overall asymmetry index); in Study 2a, the interaction was positive, suggesting that male target showed lower inversion effects when controlling for bust asymmetry; and in Study 3, no significant interaction was found.

**Target orientation * Target sex effect, controlling for bust asymmetry**

| **Study 1** | *b* = - 0.24 | *SE* = 0.12 | *p* = .06 |
| --- | --- | --- | --- |
| **Study 2a** | *b* = 0.37 | *SE* = 0.18 | *p* = .049 |
| **Study 2b** | *b* = 0.10 | *SE* = 0.21 | *p* =.62 |

In sum, also when the analysis is restricted to the “areas of interest” from the sexualization viewpoint, results show no obvious indication that asymmetry plays a consistent role in the inversion effect.
