# Supplementary material for: Measuring objectification through the Body Inversion Paradigm: Methodological issues
Source: PLoS One. 2020 Feb 19;15(2):e0229161. doi: 10.1371/journal.pone.0229161 (PMC7031944; doi:10.1371/journal.pone.0229161)
Supplement: S6 File — (DOCX) [file pone.0229161.s006.docx]

**S6. Ancillary Analysis: Bayesian meta-analytical aggregation
of the results of Study 1 and Study 2a**

Following the request of a reviewer, we conducted a Meta-Analysis, with Bayesian statistics, for the results of Study 1 and Study 2a, using the R package bmeta (Ding & Baio, 2016). This aimed at increasing sample size and, hence, the robustness of the results, providing a more comprehensive view concerning moderation hypotheses.

The following table reports the two original effects from Study 1 and Study 2a^^[[1]](#footnote-1)^^, and the mean and standard deviation for each aggregated effect, together with the Credible Interval built around the Bayesian aggregated values.

The Credible Intervals indicated that, when data of the two studies were pooled together, the moderation of target sex was disconfirmed (H1 rejection), while it was confirmed that male and female participants showed the same pattern of results (H1b confirmed). Importantly, none of the moderation effects was confirmed with the aggregated data, as none of the 95% Credible Intervals excludes the zero: On the contrary, they are all rather balanced around zero.

Table 1. Bayesian aggregation of the effects of Study 1 and Study 2a.

| **Hypotheses** | | **Study 1** | | **Study 2a** | **Aggregated effect (SD)**  **95% CrI** |
| --- | --- | --- | --- | --- | --- |
| Effect of target orientation | | *b* = 0.50 (0.06), | | *b* = 0.66 (0.09) | 0.55 (0.05), [0.45, 0.64] |
| **H1: stronger inversion for male targets** | | | | | |
| Target orientation * target sex interaction | | *b* = -0.26 (0.13) | | *b* = 0.21 (0.18) | -0.10 (0.10), [-0.31, 0.11] |
| **H1b: participants’ gender does not moderate the**  **Target orientation * the target sex interaction** | | | | | |
| Target orientation * target sex * participant gender interaction | | *b* = 0.35 (0.26) | | *b* = -0.26 (0.35) | 0.14 (0.20), [-0.27, 0.53] |
| **H2 – lower inversion for more asymmetrical stimuli** | | | | | |
| Target orientation * asymmetry interaction | *b* = -0.07 (0.07) | | | *b* = 0.23 (0.10) | 0.03 (0.06), [-0.09, 0.14] |
| **H3: lower inversion for more sexualized photos** | | | | | |
| Target orientation * sexualization interaction | *b* = -0.04 (0.06) | | | *b* =0.15 (0.10) | 0.01 (0.05), [-.09, 0.11] |
| Target orientation * sexualization * target sex interaction | *b* = -0.11 (0.13) | | | *b* =0.25 (0.30) | -0.05 (0.12), [-0.28, 0.18] |
| **H4: higher inversion for more attractive photos** | | | | | |
| Target orientation * attractiveness interaction | *b* =-0.07 (0.06) | | | *b* =0.14 (0.08) | 0.01 (0.05), [-0.09, 0.10] |
| Target orientation * attractiveness * target sex interaction | *b* = 0.35 (0.14) | | | *b* =0.08 (0.18) | 0.12 (0.11), [-0.10, 0.33] |
| **H5: lower inversion with higher SC-IAT scores** | | | | | |
| Target orientation * SC-IAT interaction | | *b* = -0.06 (0.07) | | *b* =0.01 (0.09) | -0.04 (0.06), [-0.14, 0.08] |
| Target orientation * SC-IAT * target sex interaction | | *b* = -0.06 (0.14) | | *b* =0.21 (0.18) | 0.04 (0.11), [-0.19, 0.26] |
| **H6: higher inversion for high self-objectifying participants** | | | | | |
| Target orientation * BSH interaction | | *b* = 0.02 (0.06) | | *b* =-0.02 (0.09) | 0.01 (0.05), [-0.10, 0.10] |
| Target orientation * BSH* target sex interaction | | *b* = 0.22 (0.12) | | *b* =-0.26 (0.17) | 0.06 (0.10), [-0.14, 0.26] |
| Target orientation * BSV interaction | | *b* = 0.06 (0.07) | | *b* =-0.01 (0.09) | 0.03 (0.06), [-0.08, 0.14] |
| Target orientation * BSV* target sex interaction | | *b* = 0.03 (0.13) | | *b* =-0.32 (0.18) | -0.10 (0.10), [-0.30, 0.11] |
| **H7a – effects of benevolent sexism (BS)** | | | | | |
| Target orientation * BS interaction | | *b* = -0.03 (0.06) | | *b* =-0.05 (0.09) | -0.04 (0.05), [-0.14, 0.06] |
| Target orientation * BS * target sex interaction | | *b* = 0.13 (0.13) | | *b* =-0.15 (0.17) | 0.03 (0.10), [-0.18, 0.23] |
| **H7b – effects of hostile sexism (HS)** | | | | | |
| Target orientation * HS interaction | | *b* = -0.03 (0.06) | | *b* = -0.01 (0.09) | -0.02 (0.05), [-0.12, 0.07] |
| Target orientation * HS * target sex interaction | | *b* = 0.04 (0.12) | | *b* = -0.04 (0.17) | 0.01 (0.10), [-0.17, 0.20] |
| **H7c – effects of benevolent attitude toward men (BM)** | | | | | |
| Target orientation * BM interaction | | *b* = 0.03 (0.06) | | *b* = 0.03 (0.09) | 0.03 (0.05), [-0.07, 0.13] |
| Target orientation * BM * target sex interaction | | *b* = 0.05 (0.13) | | *b* = -0.06 (0.17) | 0.01 (0.10), [-0.18, 0.22] |
| **H7d – effects of hostile attitude toward men (HM)** | | | | | |
| Target orientation * HM interaction | | *b* = -0.03 (0.06) | | *b* = -0.01 (0.09) | -0.02 (0.05), [-0.12, 0.07] |
| Target orientation * HM * target sex interaction | | | *b* = 0.10 (0.13) | *b* = -0.12 (0.17) | 0.02 (0.11), [-0.19, 0.23] |

**References**

Ding, T., Baio, G. (2016). bmeta: Bayesian Meta-Analysis and Meta-Regression. R package version 0.1.2. https://CRAN.R-project.org/package=bmeta

**R syntax used in the analysis**

if (!require("bmeta")) install.packages("bmeta")

library(bmeta)

if (!require("rjags")) install.packages("rjags")

library(rjags)

########

# Inversion effect

y <-c(0.50,0.66) #here are the regression coefficients

SD<-c(0.06, 0.09)

prec<-1/SD^2

mydata<-data.frame(y,prec)

(x <- bmeta ( data = mydata , outcome ="ctns", model ="std.mv", type ="fix"))

######## H1

# target orientation * target sex

y <-c(-0.26,0.21) #here are the regression coefficients

SD<-c(0.13, 0.18)

prec<-1/SD^2

mydata<-data.frame(y,prec)

(x <- bmeta ( data = mydata , outcome ="ctns", model ="std.mv", type ="fix"))

# (H1 b) Target orientation * target sex * participant gender

y <-c(0.35,-0.26) #here are the regression coefficients

SD<-c(0.26, 0.35)

prec<-1/SD^2

mydata<-data.frame(y,prec)

(x <- bmeta ( data = mydata , outcome ="ctns", model ="std.mv", type ="fix"))

######## H2 – lower inversion for more asymmetrical stimuli

# Target orientation * asymmetry interaction

y <-c(-0.07,0.23) #here are the regression coefficients

SD<-c(0.07, 0.10)

prec<-1/SD^2

mydata<-data.frame(y,prec)

(x <- bmeta ( data = mydata , outcome ="ctns", model ="std.mv", type ="fix"))

######## H3 - lower inversion for more sexualized photos

# Target orientation * sexualization interaction

y <-c(-0.04,0.15) #here are the regression coefficients

SD<-c(0.06, 0.10)

prec<-1/SD^2

mydata<-data.frame(y,prec)

(x <- bmeta ( data = mydata , outcome ="ctns", model ="std.mv", type ="fix"))

# Target orientation * sexualization * target sex interaction

y <-c(-0.11,0.25) #here are the regression coefficients

SD<-c(0.13, 0.30)

prec<-1/SD^2

mydata<-data.frame(y,prec)

(x <- bmeta ( data = mydata , outcome ="ctns", model ="std.mv", type ="fix"))

######## H4: higher inversion for more attractive photos

# Target orientation * attractiveness interaction

y <-c(-0.07,0.14) #here are the regression coefficients

SD<-c(0.06, 0.08)

prec<-1/SD^2

mydata<-data.frame(y,prec)

(x <- bmeta ( data = mydata , outcome ="ctns", model ="std.mv", type ="fix"))

# Target orientation * attractiveness * target sex interaction

y <-c(0.15,0.08) #here are the regression coefficients

SD<-c(0.14, 0.18)

prec<-1/SD^2

mydata<-data.frame(y,prec)

(x <- bmeta ( data = mydata , outcome ="ctns", model ="std.mv", type ="fix"))

######## H5: lower inversion with higher SC-IAT scores

# Target orientation * SC-IAT interaction

y <-c(-0.06,0.01) #here are the regression coefficients

SD<-c(0.07, 0.09)

prec<-1/SD^2

mydata<-data.frame(y,prec)

(x <- bmeta ( data = mydata , outcome ="ctns", model ="std.mv", type ="fix"))

# Target orientation * SC-IAT * target sex interaction

y <-c(-0.06,0.21) #here are the regression coefficients

SD<-c(0.14, 0.18)

prec<-1/SD^2

mydata<-data.frame(y,prec)

(x <- bmeta ( data = mydata , outcome ="ctns", model ="std.mv", type ="fix"))

######## H6: higher inversion for high self-objectifying participants

# Target orientation * BSH interaction

y <-c(0.02,-0.02) #here are the regression coefficients

SD<-c(0.06, 0.09)

prec<-1/SD^2

mydata<-data.frame(y,prec)

(x <- bmeta ( data = mydata , outcome ="ctns", model ="std.mv", type ="fix"))

# Target orientation * BSH* target sex interaction

y <-c(0.22,-0.26) #here are the regression coefficients

SD<-c(0.12, 0.17)

prec<-1/SD^2

mydata<-data.frame(y,prec)

(x <- bmeta ( data = mydata , outcome ="ctns", model ="std.mv", type ="fix"))

# Target orientation * BSV interaction

y <-c(0.06,-0.01) #here are the regression coefficients

SD<-c(0.07, 0.09)

prec<-1/SD^2

mydata<-data.frame(y,prec)

(x <- bmeta ( data = mydata , outcome ="ctns", model ="std.mv", type ="fix"))

# Target orientation * BSV* target sex interaction

y <-c(0.03,-0.32) #here are the regression coefficients

SD<-c(0.13, 0.18)

prec<-1/SD^2

mydata<-data.frame(y,prec)

(x <- bmeta ( data = mydata , outcome ="ctns", model ="std.mv", type ="fix"))

######## H7a – effects of benevolent sexism (BS)

# Target orientation * BS interaction

y <-c(-0.03,-0.05) #here are the regression coefficients

SD<-c(0.06, 0.09)

prec<-1/SD^2

mydata<-data.frame(y,prec)

(x <- bmeta ( data = mydata , outcome ="ctns", model ="std.mv", type ="fix"))

# Target orientation * BS * target sex interaction

y <-c(0.13,-0.15) #here are the regression coefficients

SD<-c(0.13, 0.17)

prec<-1/SD^2

mydata<-data.frame(y,prec)

(x <- bmeta ( data = mydata , outcome ="ctns", model ="std.mv", type ="fix"))

######## H7b – effects of hostile sexism (HS)

# Target orientation * HS interaction

y <-c(-0.03,-0.01) #here are the regression coefficients

SD<-c(0.06, 0.09)

prec<-1/SD^2

mydata<-data.frame(y,prec)

(x <- bmeta ( data = mydata , outcome ="ctns", model ="std.mv", type ="fix"))

# Target orientation * HS * target sex interaction

y <-c(0.04,-0.04) #here are the regression coefficients

SD<-c(0.12, 0.17)

prec<-1/SD^2

mydata<-data.frame(y,prec)

(x <- bmeta ( data = mydata , outcome ="ctns", model ="std.mv", type ="fix"))

######## H7c – effects of benevolent attitude toward men (BM)

# Target orientation * BM interaction

y <-c(0.03,0.03) #here are the regression coefficients

SD<-c(0.06, 0.09)

prec<-1/SD^2

mydata<-data.frame(y,prec)

(x <- bmeta ( data = mydata , outcome ="ctns", model ="std.mv", type ="fix"))

# Target orientation * BM * target sex interaction

y <-c(0.05,-0.06) #here are the regression coefficients

SD<-c(0.13, 0.17)

prec<-1/SD^2

mydata<-data.frame(y,prec)

(x <- bmeta ( data = mydata , outcome ="ctns", model ="std.mv", type ="fix"))

######## H7d – effects of hostile attitude toward men (HM)

# Target orientation * HM interaction

y <-c(-0.03,-0.01) #here are the regression coefficients

SD<-c(0.06, 0.09)

prec<-1/SD^2

mydata<-data.frame(y,prec)

(x <- bmeta ( data = mydata , outcome ="ctns", model ="std.mv", type ="fix"))

# Target orientation * HM * target sex interaction

y <-c(0.10,-0.12) #here are the regression coefficients

SD<-c(0.13, 0.17)

prec<-1/SD^2

mydata<-data.frame(y,prec)

(x <- bmeta ( data = mydata , outcome ="ctns", model ="std.mv", type ="fix"))

1. Regression values and SDs are presented in the Tables 2 to 4 in the main paper, but they are reported also here for ease of comparison of the aggregated effects with the original data of the two studies. [↑](#footnote-ref-1)
